# Fossils of the oldest diplodocoid dinosaur suggest India was a major centre for neosauropod radiation

**DOI:** 10.1038/s41598-023-39759-2

**Published:** 2023-08-04

**Authors:** Sunil Bajpai, Debajit Datta, Pragya Pandey, Triparna Ghosh, Krishna Kumar, Debasish Bhattacharya

**Affiliations:** 1 Department of Earth Sciences, Indian Institute of Technology, Roorkee, Uttarakhand 247667 India; 2 Geological Survey of India, Raipur, Chhattisgarh 492010 India; 3 Geological Survey of India, Jaipur, Rajasthan 302004 India; 4 Central Head Quarters, Geological Survey of India, Kolkata, West Bengal 700091 India

**Keywords:** Palaeontology, Palaeontology

## Abstract

The Early Jurassic and Cretaceous deposits of India are known for their diverse sauropod fauna, while little is known from the Middle and Late Jurassic. Here we report the first ever remains of a dicraeosaurid sauropod from India, *Tharosaurus indicus* gen. et sp. nov., from the Middle Jurassic (early–middle Bathonian) strata of Jaisalmer Basin, western India. Known from elements of the axial skeleton, the new taxon is phylogenetically among the earlier-diverging dicraeosaurids, and its stratigraphic age makes it the earliest known diplodocoid globally. Palaeobiogeographic considerations of *Tharosaurus*, seen in conjunction with the other Indian Jurassic sauropods, suggest that the new Indian taxon is a relic of a lineage that originated in India and underwent rapid dispersal across the rest of Pangaea. Here we emphasize the importance of Gondwanan India in tracing the origin and early evolutionary history of neosauropod dinosaurs.

## Introduction

Sauropods, a speciose group of saurischian dinosaurs that dominated the terrestrial landscapes until the end-Cretaceous^1,2^, are characterized by a small skull, elongated neck and tail, columnar limbs, and a quadrupedal gait^3^. Within Sauropoda, Dicraeosauridae represents a clade of small-bodied diplodocoids that are known for their distinctive vertebral morphology with long paired neural spines^4–7^. Dicraeosaurids range in age from the Middle Jurassic–Early Cretaceous and are mostly known from the Gondwanan landmasses of Africa and South America, besides a few Laurasian occurrences in the USA and China^3,8^. Amongst the well-known dicraeosaurids are *Dicraeosaurus* from East Africa and *Brachytrachelopan*, *Amargasaurus*, *Bajadasaurus*, *Pilmatueia* and *Amargatitanis* from Argentina^4,5,8–11^, *Suuwassea*, *Kaatedocus* and *Smitanosaurs* from USA^12^, and *Lingwulong* from China^13^.

**Figure 1:**
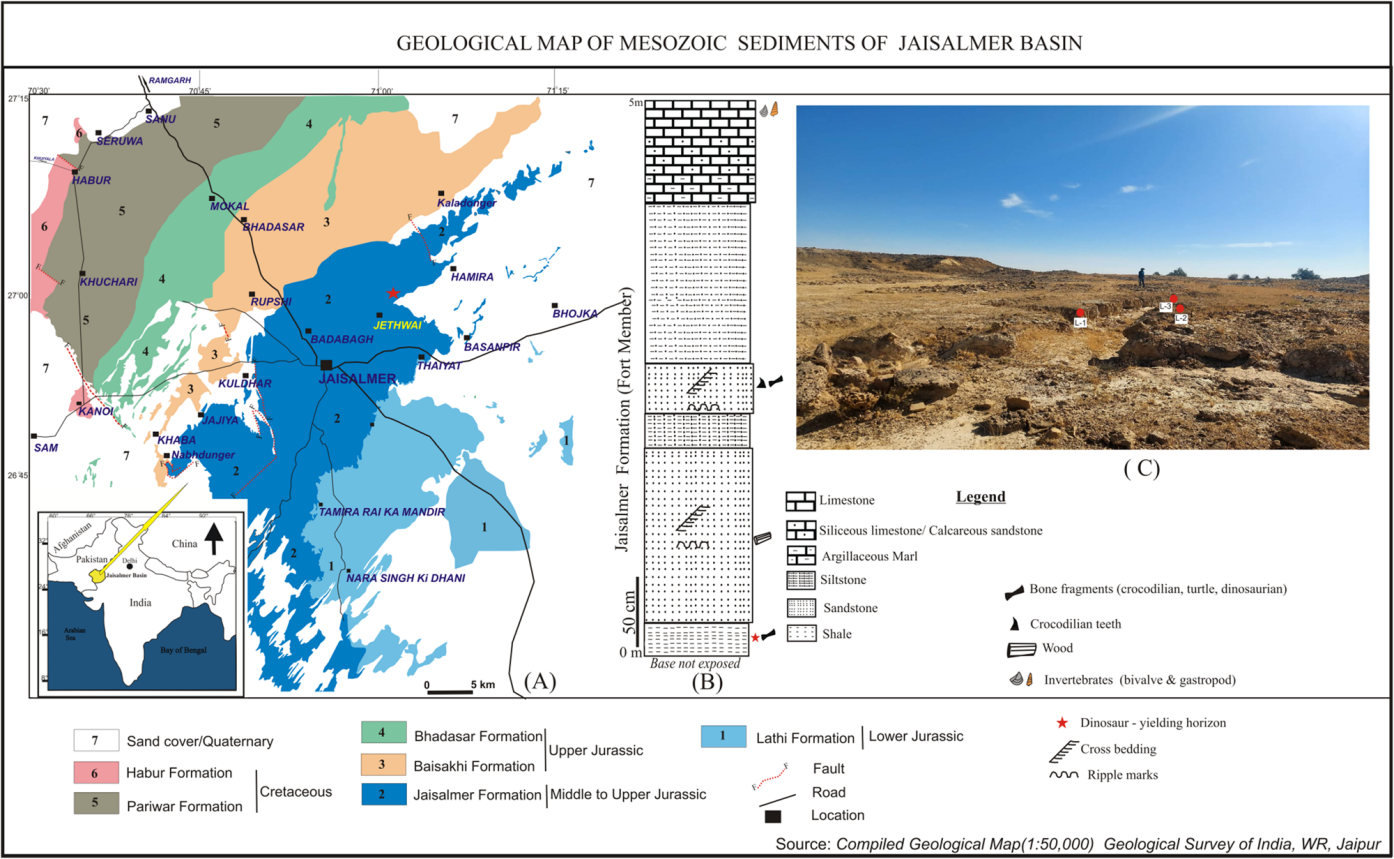
Geological map of Jaisalmer Basin showing (**a**) the fossil locality; (**b**) stratigraphic column showing the position of the dinosaur fossil yielding horizon; (**c**) photograph of the fossil site. The map and stratigraphic column were drawn by K.K. using CorelDRAW 2019 (Version number: 21.0.0.593, URL link: http://www.corel.com/en/).

In India, early-diverging sauropods *Barapasaurus* and *Kotasaurus* are known from the Early Jurassic Kota Formation of Pranhita–Godavari Basin, whereas putative Middle Jurassic camarasauromorph remains occur in Kutch^2,14–18^. No diplodocoid sauropods are yet known from India^18,19^. Here we report on the discovery of a new dicraeosaurid from the Jaisalmer Formation, Rajasthan, western India (Fig. 1, Supplementary Note 1). Fossils were collected from a shale unit situated at the base of the early–middle Bathonian Fort Member^20,21^ and include disarticulated, but associated, specimens of the axial skeleton spread over an area of ~ 25 m^2^ (L1–L3, Fig. 1). This discovery provides new insights into sauropod diversity of the Indian Gondwana, with important implications for the origin and dispersal of Neosauropoda.

## Results

### Systematic palaeontology

Sauropoda Marsh, 1878

Neosauropoda Bonaparte, 1986

Diplodocoidea Marsh, 1884

Dicraeosauridae Janensch, 1929

*Tharosaurus indicus* gen. et sp. nov.

#### Etymology

Generic name is a combination of *Tharo*, referring to the ‘Thar desert’ of western India where the type specimen was found, and *saurus*, which is derived from the Greek word ‘sauros’ meaning lizard; specific name is for the country of origin i.e., India.

#### Holotype

**Figure 2:**
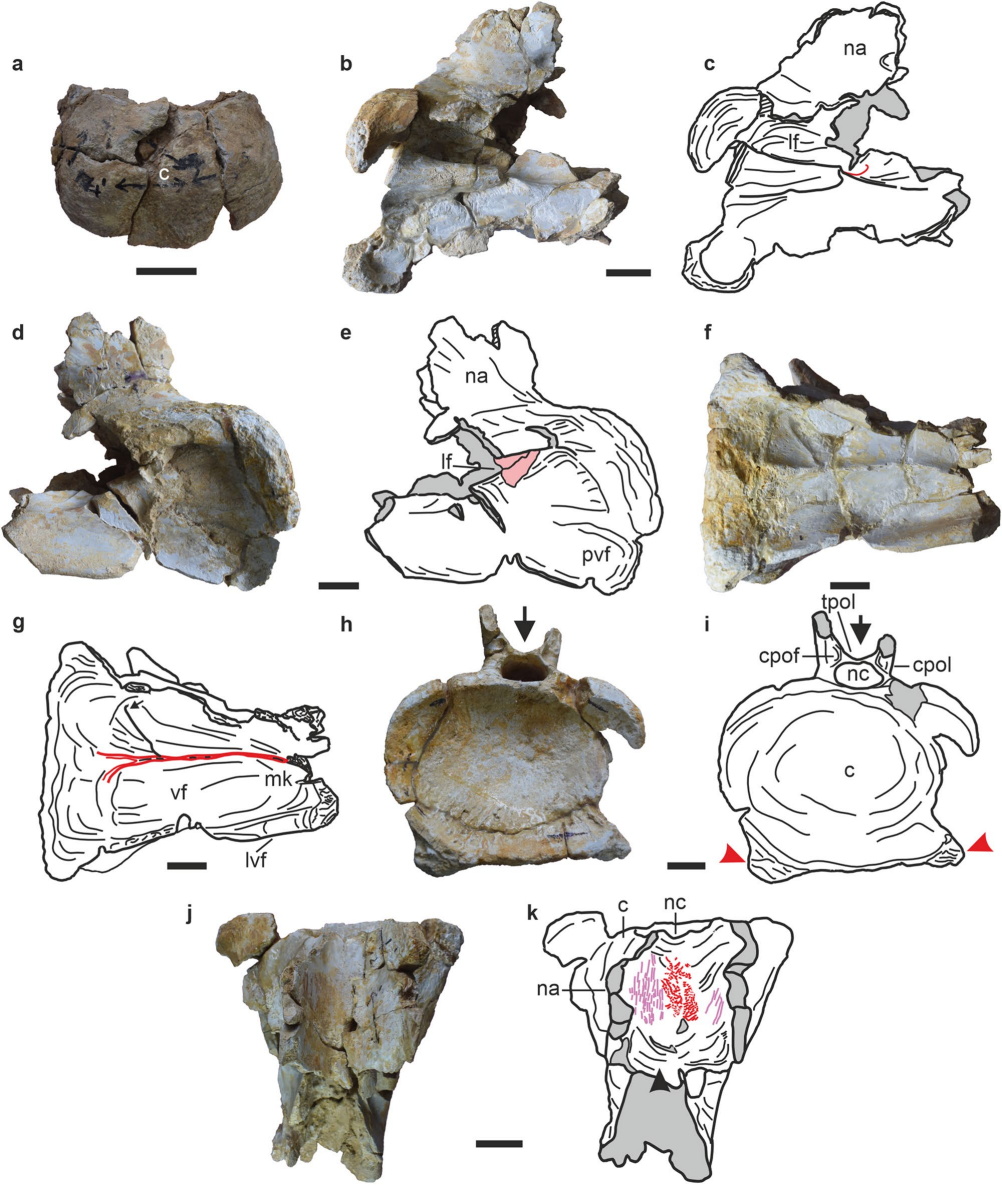
Cervical vertebrae (CV6/8) of *Tharosaurus indicus*. (**a**) RWR-241-A, anterior cotyle in anterior view. (**b**–**k**) RWR-241-B, partial vertebra, photographs and line drawings in (**b**,**c**) right lateral view, red line indicates U-shaped ridge demarcating anterior and posterior halves of lateral pneumatic fossa; (**d**,**e**) left lateral view; (**f**,**g**) ventral view, red line indicates posteriorly bifurcated midline keel and arrow indicates accessory ridge; (**h**,**i**) posterior view, arrows and red arrowheads indicate deep bifurcation of neural arch and triangular facets below cotyle, respectively. (**j**,**k**) dorsal view, arrowhead indicates passage enclosed by bifid neural arch and ligament scars and striations marked in red and purple, respectively. Broken areas and artefacts in grey and pink, respectively. *c* centrum, *cpof* centropostzygapophyseal fossa, *cpol* centropostzygapophyseal lamina, *lf* lateral fossa, *lvf* lateroventral flange, *mk* midline keel, *na* neural arch, *nc* neural canal, *pvf* posteroventral fossa, *tpol* intrapostzygapophyseal lamina. Scale bars represent 50 mm.

**Figure 3:**
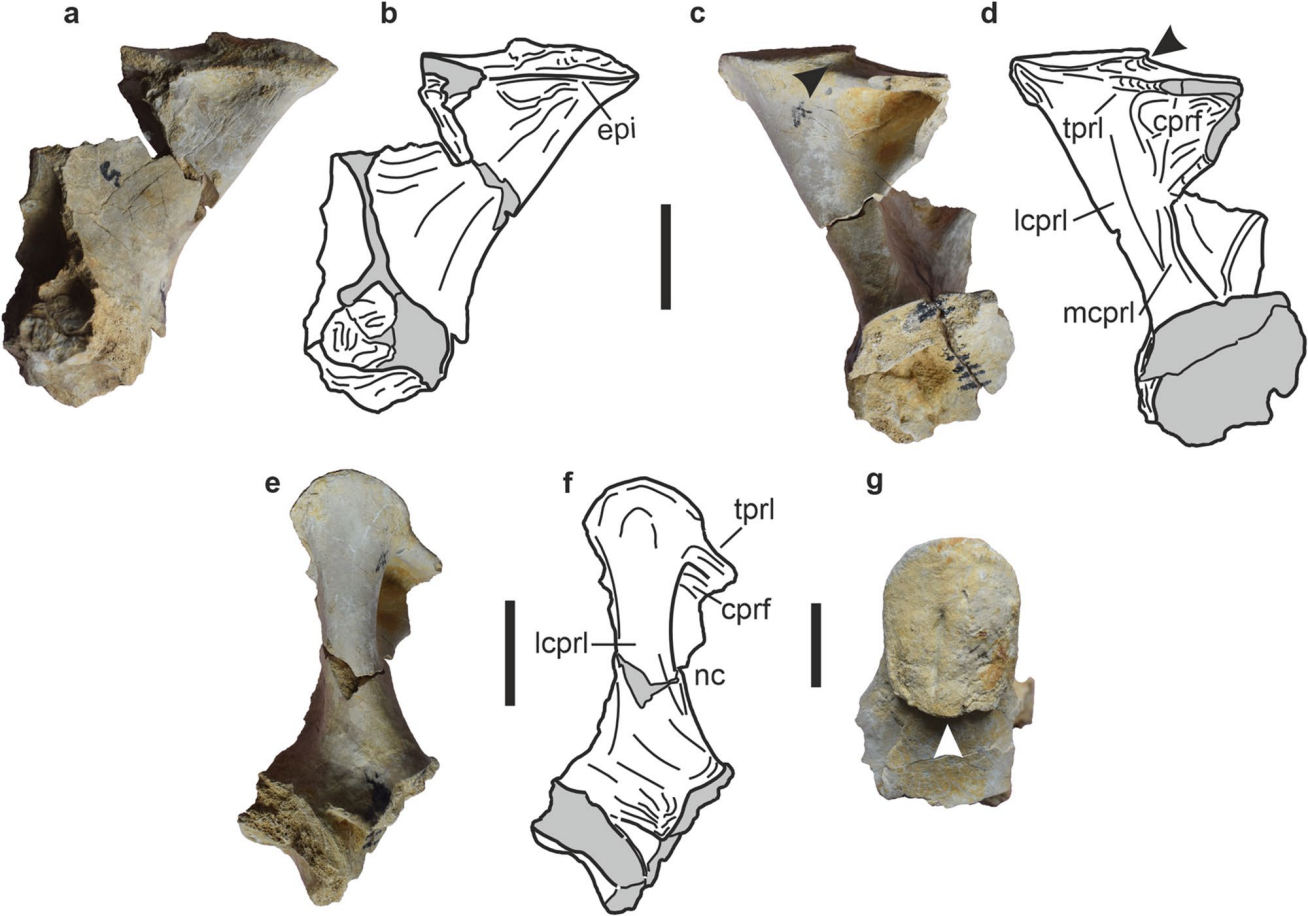
Right cervical prezygapophysis (CV6/8) of *Tharosaurus indicus*. RWR-241-C, photographs and line drawings in (**a**,**b**) lateral view; (**c**,**d**); medial view; (**e**,**f**) anterior view; (**g**) dorsal view. Arrowheads indicate transverse sulcus beneath dorsal articular surface of prezygapophysis. *cprf* centroprezygapophyseal fossa, *epi* pre-epipophysis, *lcprl* lateral branch of centroprezygapophyseal lamina, *mcprl* medial branch of centroprezygapophyseal lamina, *nc* neural canal, *tprl* intraprezygapophyseal lamina. Scale bars represent 50 mm.

**Figure 4:**
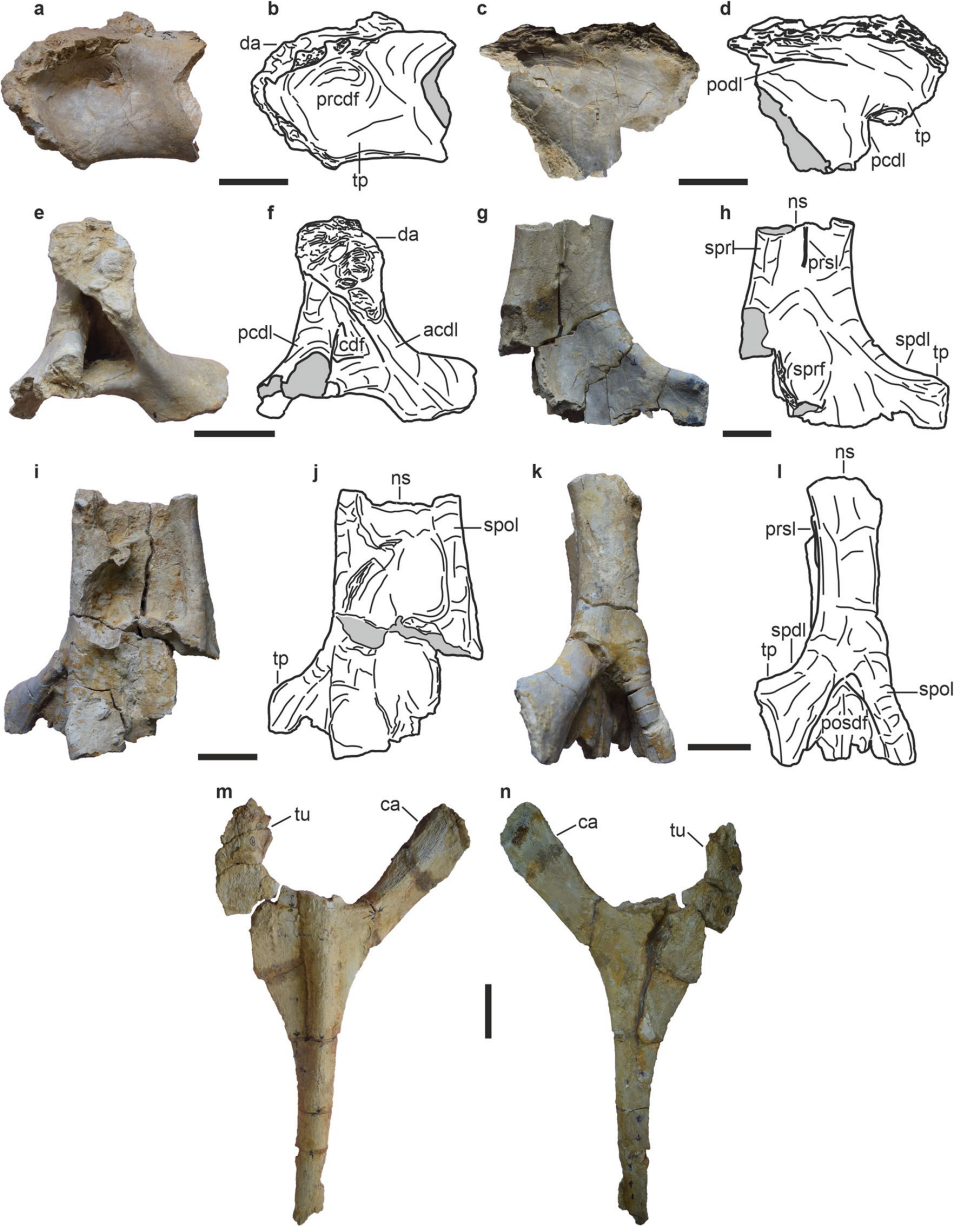
Dorsal vertebrae of *Tharosaurus indicus*. RWR-241-F, partial anterior dorsal neural arch, photographs and line drawings in (**a**,**b**) anterior view; (**c**,**d**) posterior view; (**e**,**f**) anterior view. RWR-241-G, partial middle/posterior dorsal neural arch-spine complex, photographs and line drawings in (**g**,**h**) anterior view; (**i**,**j**) posterior view; (**k**,**l**) lateral view. RWR-241-I, nearly complete anterior dorsal rib in (**m**) anterior view; (**n**) posterior view. *acdl* anterior centrodiapophyseal lamina, *ca* capitulum, *cdf* centrodiapophyseal fossa, *da* diapophysis, *ns* neural spine, *pcdl* posterior centrodiapophyseal lamina, *podl* postzygodiapophyseal lamina, *posdf* postzygapophyseal spinodiapophyseal fossa, *prcdf* prezygapophyseal centrodiapophyseal fossa, *prsl* prespinal lamina, *spdl* spinodiapophyseal lamina, *spol* spinopostzygapophyseal lamina, *sprl* spinoprezygapophyseal lamina, *sprf* spinoprezygaposphyseal fossa, *tp* transverse process, *tu* tuberculum. Scale bars represent 50 mm.

**Figure 5:**
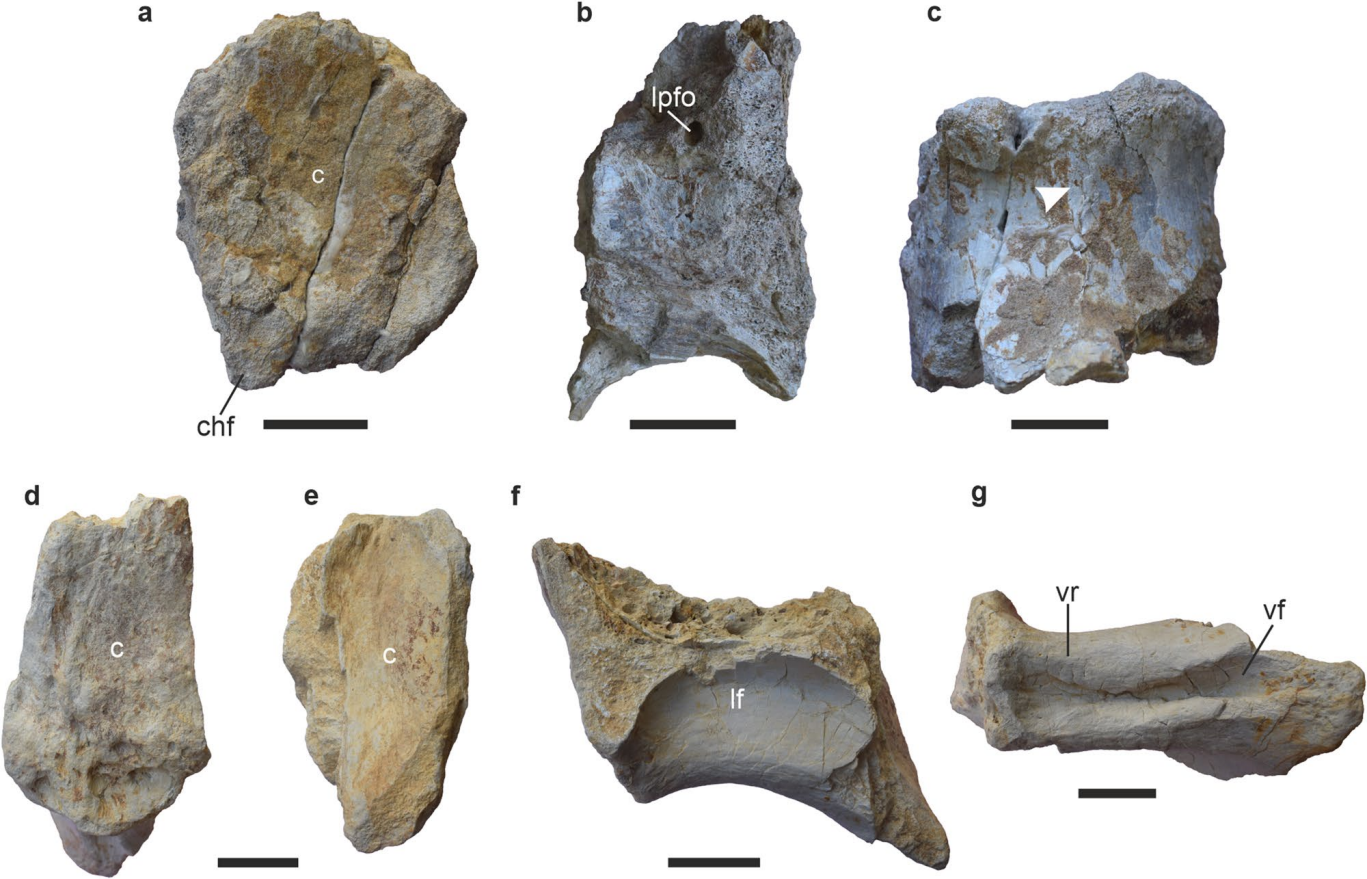
Caudal vertebrae of *Tharosaurus indicus*. RWR-241-J, partial anterior caudal vertebra in (**a**) anterior view; (**b**) right lateral view; (**c**) ventral view. RWR-241-K, middle caudal centrum in (**d**) anterior view; (**e**) posterior view; (**f**) left lateral view; (**g**) ventral view. *c* centrum, *chf* chevron facet, *lf* lateral fossa, *lpfo* lateral pneumatic foramen, *vf* ventral fossa, *vr* ventrolateral ridge.

RWR-241A–K (Palaeontology Division, Geological Survey of India, Western Region, Jaipur, Rajasthan, India); partial middle/posterior cervical vertebra; middle/posterior cervical anterior condyle and right prezygapophyses; partial anterior dorsal neural arch; middle/posterior neural spines; anterior dorsal rib; partial anterior and middle caudal vertebrae (Figs. 2, 3, 4, 5; Supplementary Table 1).

#### Horizon and locality

Fort Member of the Jaisalmer Formation (early–middle Bathonian), Jethwai village, District Jaisalmer, Rajasthan state, western India.

#### Diagnosis

*Tharosaurus* exhibits a unique combination of the following characters: middle/posterior cervical centroprezygapophyseal lamina divided into lateral and medial branches, the latter connecting with the intraprezygapophyseal lamina (shared with all dicraeosaurids); middle/posterior cervical centropostzygapophyseal fossa elliptical and bordered laterally by pillar-like centropostzygapophyseal lamina (shared with the dicraeosaurids *Lingwulong*, *Brachytrachelopan*, and *Pilmatueia*); deep bifurcation of cervical neural arch extending up to the dorsal margin of the neural canal (shared with the dicraeosaurids *Amargasaurus* and *Pilmatueia*); paired fossae on the ventral surface of the middle/posterior cervical centrum separated by a mid-line keel (shared with all dicraeosaurids, barring *Smitanosaurus*, *Kaatedocus* and *Suuwassea*, and the diplodocids—*Barosaurus* and *Dinheirosaurus*); paired ventral fossae extending up to the posterior margin of centrum; anterior condyle of middle/posterior cervical more rugose compared to the rest of the centrum (shared with the dicraeosaurid *Kaatedocus*); bifid middle/posterior cervical neural spine (shared with Flagellicaudata); divided lateral fossa/pleuroceol on cervical centra (shared with Flagellicaudata); lateral fossa divided into deep posterior and shallow anterior half by weak ridge on the ventral surface of the fossa; prominent lateroventral flanges on middle/posterior cervical centrum (shared with Flagellicaudata); middle/posterior cervical vertebrae with pre-epipophysis (shared with Flagellicaudata); cordiform cross-section of anterior caudal centra (shared with Flagellicaudata); middle/posterior dorsal transverse process laterally directed (shared with Diplodocidae); middle caudal centra articular surface with flat ventral margin (shared with Diplodocoidea); ventral surface of mid-caudal centrum with deep median fossa.

Autapomorphies: triangular ventrolateral projections lying below posterior cotyle of middle/posterior cervical centrum visible in posterior view; ventral mid-line keel on middle/posterior cervical centrum bifurcating posteriorly but not meeting the lateroventral flanges; lateral pleuroceol on anterior caudal centra.

### Description

The cervical vertebrae are represented by five partial specimens found in association (Supplementary Table 1), including the anterior condyle, posterior half of a vertebra and three right prezygapophyses (Figs. 2, 3). The anterior condyle is U-shaped with an opisthocoelous condition as in other eusauropods^22,23^ (Fig. 2A), and is mediolaterally wider than dorsoventrally tall [RWR-241-A, centrum articular surface height (cH)/centrum articular surface width (cW) = 0.7; Supplementary Table 2, Supplementary Fig. 2]. In this respect, *Tharosaurus* is similar to the middle/posterior cervicals of several flagellicaudatans: *Suuwassea*^24^ (cH/cW = 0.7), *Lingwulong*^13^ (cH/cW = 0.7), *Pilmatueia*^8^ (CV7/8, cH/cW = 0.9), *Amargasaurus*^5^ (CV8, cH/cW = 0.8), *Apatosaurus louisae*^25^ (CV9, cH/cW = 0.7) and *Diplodocus carnegii*^26^ (CV11, cH/cW = 0.9). The anterior condyle is markedly more rugose than the other vertebral elements, as in *Kaatedocus*^27^ (CV14).

The centrum shows strong anterior constriction with the lateral surfaces excavated by large fossae, similar to many flagellicaudatans^8,13,24,26^ (Fig. 2B–E). The fossa is partially preserved with much of the dorsal rim and medial wall broken off. The preserved portion suggests the fossa was elliptical, being elongated and dorsoventrally compressed (Fig. 2B–C). The dorsal margin of the fossa is rod-like and extends laterally beyond the ventral margin. The latter is robust, rounded and thickest near the posterior end of the centrum, accentuating the depth of the fossa. Anteriorly it becomes flush with the lateral surface of the centrum (Fig. 2B–C). Beneath the ventral margin of the fossa, the lateral surface of the centrum is markedly concave and flares posteriorly. The fossa is restricted to the posterior half of the centrum and includes two distinct halves: a short and shallow anterior half and a longer and deeper posterior half. The two halves are demarcated by a low U-shaped ridge on the ventral surface of the fossa, medial to the ventral margin and extending slightly onto the medial wall of the fossa. This contrasts with the dicraeosaurids *Amargasaurus*, *Dicraeosaurus*, *Bajadasaurus*, and *Pilmatueia* where the fossa is undivided and shallow^5,7,8^. In the middle cervicals of *Lingwulong* and *Suuwassea*, however, the lateral fossa is deep and extends nearly along the entire length of the centrum^13,24^. Furthermore, the lateral fossa in *Tharosaurus* is succeeded by a posteroventral fossa (Fig. 2D,E), as seen in the mid-cervicals of *Amargasaurus*, *Apatosaurus*, *Bajadasaurus*, and *Pilmatueia*^26,28^.

The ventral surface of the centrum (Fig. 2F–G) accommodates paired longitudinal depressions/fossae. Such depressions are common in diplodocids and most dicraeosaurids, barring *Bajadasaurus* which only bears a longitudinal keel^7,8,13,26^. The fossae are asymmetrical, with the right fossa mediolaterally wider, and extend up to the posterior margin of the centrum where they flare out. However, in *Dicraeosaurus*, *Lingwulong*, and *Pilmatueia* the fossae are symmetrical and strongly expressed anteriorly, but do not reach the posterior margin of the centrum^8,13,26^. The fossae in *Tharosaurus* are flanked laterally by prominent lateroventral flanges and separated by a sharp midline keel (Fig. 2F,G). The keel shows left lateral convexity and remains prominent throughout its preserved length. It extends nearly up to the posterior margin of the fossae and bifurcates into two short ridges. The latter, however, do not reach up to the lateroventral flanges. An accessory ridge, present only on the right ventral surface, extends posterolaterally from the midline keel. The presence of a ventral keel is shared with a diverse array of flagellicaudatans including most dicraeosaurids (*Lingwulong*, *Dicraeosaurus*, *Pilmatueia*, *Bajadasaurus* and *Brachytrachelopan*)^7–9,13^ and some later-diverging diplodocids (*Barosaurus lentus*, *Diplodocus carnegii*, *Dinheirosaurus*)^23,26^. It is, however, absent in some putative plesiomorphic dicraeosaurids (*Suuwassea* and *Smitanosaurus*)^24,29^, barring *Kaatedocus*^27^ which is the earliest-diverging dicraeosaurid. The disposition and morphology of this midline keel differentiate *Tharosaurus* from *Lingwulong*, *Dicraeosaurus*, *Brachytrachelopan* and *Barosaurus* where the keel is prominent anteriorly^4,8,9,26^. In *Dinheirosaurus* the keel is restricted to the posterior part of the centrum and does not extend anteriorly as in *Tharosaurus*, whereas in *Pilmatueia* the keel forks anteriorly and posteriorly with the bifurcation point placed slightly anterior to the mid-length of the centrum.

The posterior cotyle of the centrum (RWR-241-B, cH/cW = 0.6, Fig. 2H,I, Supplementary Table 2) is strongly concave and mediolaterally wider than dorsoventrally tall, a feature seen in the middle cervicals of many sauropods including *Lingwulong*^13^ (cH/cW = 0.7), *Apatosaurus louisae*^25^ (cH/cW = 0.7) and *Gleamopus*^30^ (cH/cW = 0.9), and the posterior cervicals of *Pilmatueia*^8^ (cH/cW = 0.6) and *Suuwassea*^24^ (cH/cW = 0.7). The posterior cotyle is cordiform in outline with a weakly concave dorsal margin immediately ventral to the neural canal as in *Lingwulong* and *Pilmatueia*^8,13^. *Tharosaurus*, however, can be distinguished by the presence of posteriorly facing triangular facets along the ventrolateral margin of the posterior articular surface.

The neural arch is partially preserved and includes the right prezygapophysis and the region surrounding the neural canal (Figs. 2B–K, 3, Supplementary Table 1). The anterior exit of the canal, although partial, appears oval, but the posterior exit is complete and elliptical, being transversely wider than dorsoventrally high. In *Pilmatueia* this exit is triangular, whereas in *Suuwassea* and *Lingwulong* it is circular–subcircular^8,13,24^.

The prezygapophysis is anterodorsally directed with the articular facet strongly convex transversely and longitudinally (Fig. 3A–G). The latter feature is shared with middle and posterior cervicals of the flagellicaudatans *Diplodocus carnegii* (CV8) and *Kaatedocus* (CV10)^26–29^. The prezygapophyseal articular surface is oblong with the posterior border offset from the surrounding dorsal surface of the prezygapophysis by a transverse sulcus (Fig. 3C,D,G), similar to the posterior cervicals of *Kaatedocus*^27^. Immediately ventral to the articular surface, the lateral surface of the prezygapophysis bears an anterodorsally-posteroventrally oriented pre-epipophysis (sensu Tschopp et al.^26^; Fig. 3A,B). The latter is dorsoventrally compressed and succeeded ventrally by a shallow fossa. Such a ridge is also reported in the middle and posterior cervicals of *Kaatedocus*^27^ (CV7–10), *Apatosaurus louisae*^26^ (CV11) and *Suuwassea*^24,26^. The prezygapophysis is supported ventrally by a dorsoventrally high and robust centroprezygapophyseal lamina (cprl, Fig. 3C–F). The lamina is divided into a larger and medially concave lateral branch connecting with the prezygapophysis (lcprl) and a smaller medial branch joining the intraprezygapophyseal lamina (mcprl). The two halves of the cprl accommodate a triangular and deep centroprezygapophyseal fossa (Fig. 3C,D) which borders the neural canal dorsolaterally and is roofed by a sheet-like intraprezygapophyseal lamina. While a divided cprl is common in Flagellicaudata^1^, in dicraeosaurids the medial branch of the cprl connects with the intraprezygapophyseal lamina and is listed as a synapomorphy of Dicraeosauridae (sensu Whitlock and Wilson Mantilla^29^). Unlike in *Tharosaurus*, however, both branches of the cprl join the prezygapophysis in diplodocids^1,26,29,31^. Nonetheless, the anterior surface of the lcprl immediately ventral to the prezygapophyseal articular surface bears a depression giving the impression of a split cprl, where both branches connect to the prezygapophysis, akin to diplodocids. Further scrutiny shows this depression to be almost indistinct and unlike the morphology of the bifurcated crpl of diplodocids^26,32^. Furthermore, in diplodocids the apex of this bifurcation is ventrally directed in contrast to the incipient depression in *Tharosaurus* which is broad and dorsally convex. Therefore, the expression of the divided cprl in the new Indian taxon is closer to that of dicraeosaurids and supports its taxonomic allocation.

The posterior exit of the neural canal is flanked dorsolaterally by two elliptical centropostzygapophyseal fossae [cpof(e)] which are bordered laterally by robust pillar-like centropostzygapophyseal laminae [cpol(e), Fig. 2H,I]. The cpof(e) are roofed by thin intrapostzygapophyseal laminae. The arrangement of the vertebral laminae and fossae in *Tharosaurus* is comparable with the condition in *Lingwulong*, *Brachytrachelopan*, *Dicraeosaurus*, *Amargasaurus* and *Pilmatueia*^4,5,8,9,13^. However, in the latter two taxa, the cpof(e) are triangular. Furthermore, as in *Amargasaurus*^5,8^, the neural arch in *Tharosaurus* is devoid of a median tubercle.

The preserved neural arch above the neural canal shows deep bifurcation which descends to the roof of the canal and encloses an anteroposteriorly extensive passage of uniform width (Fig. 2H–K; Supplementary Fig. 3). The broken ends of the neural arch dorsal to the cpof(e) suggest the presence of strongly divergent postzygapophyses. These features are reminiscent of the deeply bifurcated neural arch-spine complex of middle to cervicodorsal vertebrae of *Amargasaurus* and *Pilmatueia*^5,8,28^. Moreover, the surface of the neural arch within this passage is finished (as seen in dorsal view, Fig. 2J,K), and bears rugose scars along the mid-line which possibly represent ligament scars. The scars largely occupy the posterior half of the passage and bordered laterally by sub-parallel striations. Rugose tuberosity on the floor of bifurcated neural arch-spine complexes of *Apatosaurus ajax* and *Dicraeosaurus* have been interpreted as ligament scars^33^. Although *Tharosaurus* lacks the tuberosity, a feature shared with *Amargasaurus*^8^, these scars attest to the presence of bifid neural spines in *Tharosaurus*. Similar bifid neural spines are listed as a synapomorphy of Flagellicaudata in previous studies (e.g., Wilson^1^).

The specimens (RWR-241-A–E) likely represent the 6th/8th cervical based on comparisons with the middle cervicals of *Lingwulong* and *Bajadasaurus*, and the middle–posterior cervicals of *Dicraeosaurus*, *Suuwassea*, *Amargasaurus*, *Pilmatueia* and *Kaatedocus*^5,8,13,24,27^.

Three isolated specimens including a partial neural arch and two partial neural arch-spine complexes are referable to the dorsal vertebral series (Fig. 4A–L, Supplementary Table 1). The neural arch comprises a stout, sub-horizontal transverse process with an anteroposteriorly broad and sigmoidal diapophysis (Fig. 4A–F). Anteriorly, the transverse process bears a broad and moderately deep prezygapophyseal centrodiapophyseal fossa (Fig. 4A,B), whereas its posterior surface bears a thin, mediodorsally-lateroventrally directed lamina, possibly representing the postzygodiapophyseal lamina (Fig. 4C,D). A deep centrodiapophyseal fossa (cdf) positioned ventral to the transverse process is bordered by an anteroventrally directed anterior centrodiapophyseal lamina and a near-vertical posterior centrodiapophyseal lamina (Fig. 4E,F). The specimen is tentatively assigned to the anterior dorsals based on similarity in overall morphology including laminae and fossae configuration with those of the flagellicaudatans *Apatosaurus louisae*, *Dinheirosaurus*, *Lingwulong* and *Amargasaurus cazaui*^5,13,23,25,26^.

The neural arch-spine complex is strongly compressed anteroposteriorly and expanded transversely, suggesting a more posterior position in the dorsal vertebral series (sensu McPhee et al.^34^). The arch comprises a transversely broad and subtriangular spinoprezygapophyseal fossa (Fig. 4G,H). The transverse process is laterally directed, with a vertically oriented distal end, similar to all diplodocids^23,35^. This laterally oriented transverse process can be considered a local autapomorphy of *Tharosaurus* unlike a dorsolaterally oriented transverse process in other dicraeosaurids^5,9,13,36^. This feature may alternatively represent a symplesiomorphy, although Mannion et al.^23^ listed the dorsolaterally projecting diapophysis in the dorsal vertebrae of the diplodocid *Dinheirosaurus* as a local autapomorphy. The neural spine is transversely expanded and non-bifid and the disposition of the lateral spinal margins suggests dorsal flaring in anterior/posterior views (Fig. 4G–J). This suggests affinity with the middle/posterior dorsal vertebrae because non-bifurcated neural spines appear from the 6th/7th dorsal of dicraeosaurids, as seen in *Lingwulong* and *Brachytrachelopan*^9,13^. Such flaring of the lateral spinal margins is also known from the middle and posterior dorsals of dicraeosaurids^4,13,35^. In lateral view, the anterior and posterior borders of the neural spine remain subparallel (Fig. 4K,L), unlike the sub-triangular spinal lateral profile characteristic of many titanosauriforms^34,37^. The neural spine bears a sharp prespinal lamina placed along the longitudinal midline on the anterior surface (Fig. 4G,H). The prespinal lamina does not extend up to the base of the spine and terminates dorsal to the spinoprezygapophyseal fossa. This lamina, however, extends down to the base of the neural spine in the posterior dorsals of *Amargasaurus* and *Dicraeosaurus*^5,36^. Only a small part of the spinoprezygapophyseal lamina is preserved along the lateral margin of the anterior spinal surface (Fig. 4G,H). The spinodiapophyseal lamina is robust and broad, descending gently from the neural spine to the transverse process (Fig. 4G,H,K,L). The posterior surface of the spine preserves only the right spinopostzygapophyseal lamina (Fig. 4I,J). It is robust and well-rounded, extending medially from the lateral margin of the posterior spinal surface towards the midline. In lateral view, the anterior surface of the spinopostzygapophyseal lamina unites with the posterior surface of the spinodiapophyseal lamina as seen in the posterior dorsals of *Brachytrachelopan*^9^ (Fig. 4K,L), and these laminae enclose the postzygapophyseal spinodiapophyseal fossa. Thus, these vertebral specimens likely belong to the middle/posterior dorsals.

An isolated, nearly complete rib (Fig. 4M,N) is identified as the right anterior dorsal rib, based on similarity with that of *Galeamopus pabsti*^30^. The specimen is Y-shaped, flares out proximally, and becomes dorsoventrally skewed distally. The rib lacks pneumatic openings as in diplodocoids but contrasts with those of titanosauriforms^38^. The capitulum is elliptical, anteroposteriorly compressed, and directed anterodorsally. It is longer than the tuberculum and bears prominent striations on the anterior surface (Fig. 4M). The tuberculum is short and curved towards the capitulum. The tuberculum and capitulum enclose a broad U-shaped space. A robust and rounded ridge occupies the anterior surface of the rib distal to the capitulum and tuberculum (Fig. 4M). The ridge extends distally and gradually becomes flush with the surface. The ventral margin is sigmoidal with the portion immediately distal to the tuberculum strongly convex. The dorsal margin is concave, and its posterior surface is largely flat and featureless, except for the strongly rugose tuberculum.

The collection includes two caudal vertebrae (Supplementary Table 1). One of these, a partial centrum of an anterior caudal vertebra, preserves the anterior cotyle and the proximal-most part of the neural arch (Fig. 5A–C). The centrum is robust with a moderately concave anterior cotyle. The latter is dorsoventrally taller than mediolaterally wide (RWR-241-J, cH/cW = 1.2, Supplementary Table 2) and comparable in proportion to the anterior caudals of other flagellicaudatans such as *Dicraeosaurus*^39,40^ (cH/cW = c. 1), *Suuwassea*^24^ (ANS 21122, cH/cW = 1.1), *Lingwulong*^13^ (cH/cW = 1.1) and *Amazonsaurus*^41^ (cH/cW = 1.1). It is, however, dorsoventrally taller compared to *Diplodocus*^40^ (cH/cW = 0.9), *Amargatitanis*^11^ (MACN PV N53, cH/cW = 0.7) and *Brontosaurus excelsus*^26,42^ (YPM 1980, cH/cW = 0.9). The ventral margin of the anterior cotyle, although partial, preserves a small chevron facet (Fig. 5A). The preserved lateral surface of the centrum is convex and bears a lateral foramen immediately ventral to the neural arch, a feature present in anterior caudals of most diplodocids^23,40,43^ (Fig. 5B). The lateral surface, however, does not bear any ridges, similar to *Amargatitanis*^11^. In lateral view, the ventral margin of the centrum is strongly concave, akin to *Lingwulong*^13^. Moreover, the curvature of the ventral margin suggests the ventral rim of the posterior central articular surface to be at a lower level than the anterior, a feature shared with *Dicraeosaurus*^39^ and *Suuwassea*^24^. The neural arch is poorly preserved (Fig. 5A,B) and placed anteriorly on the centrum as in *Dicraeosaurus*^39^, *Lingwulong*^13^ and *Suuwassea*^24^. The ventral surface is weakly convex transversely with an incipient midline keel (Fig. 5C). This ventral keel is also present in other dicraeosaurids, including *Suuwassea* and *Dicraeosaurus*^8,24,39^.

The second caudal (RWR-241-K) vertebra is a nearly complete centrum but without the neural arch (Fig. 5D–G). The centrum is anteroposteriorly elongated, suggesting a more posterior position in the caudal series compared to RWR-241-I (sensu Coria et al.^8^). The centrum is 1.6 times as long as high, with a deep fossa extending along the entire length of the centrum, identifying it to be a middle caudal (sensu McPhee et al.^34^). This is corroborated by similarities with middle caudal centra of *Suuwassea*^24^ (cL/cH = 1.5) and *Amargatitanis*^11^ (cL/cH = 1.3). A prominent lateral fossa is also reported in the dicraeosaurid *Amargatitanis*^11^. Furthermore, judging from the broken dorsal surface of the centrum, the neural arch was possibly more anteriorly placed in RWR-241-J as in the afore-mentioned dicraeosaurids. The centrum is platyceolous with the anterior and posterior cotyles dorsoventrally taller than mediolaterally wide (Fig. 5D,E), although there is some evidence of deformation. The ventral surface is strongly concave in lateral view with the ventral margin of the posterior cotyle descending below the level of the anterior cotyle. The fossa on the ventral surface is deep, constricted at midlength and bordered by robust ventrolateral ridges (Fig. 5G), similar to the derived diplodocids such as *Diplodocus*^44^, *Seismosaurus*^45^, and *Barosaurus*^46^.

### Phylogenetic analysis

**Figure 6:**
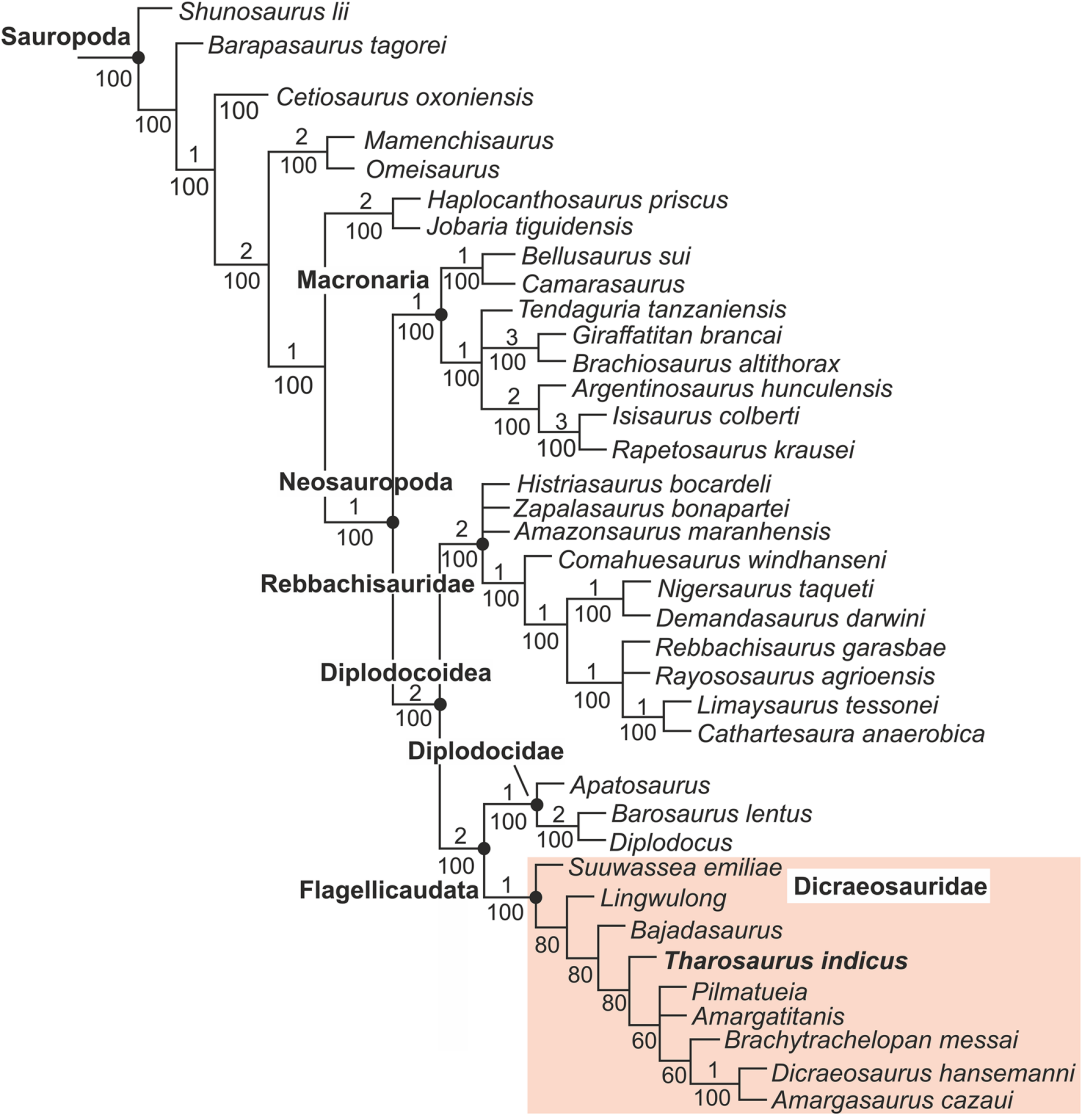
Phylogenetic position of *Tharosaurus indicus* gen. et sp. nov. (RWR-241) in 50% majority-rule tree. Clade Dicraeosauridae shaded in pink. Numbers above nodes indicate Bremer support values.

The inter-relationship of *Tharosaurus* within Sauropodomorpha was tested in a reduced version of the data matrix used by Gallina et al.^7^ (see “Methods” and Supplementary Note 2). The analysis recovered 20 most parsimonious trees with a tree length of 760, consistency index (CI) of 0.483 and retention index (RI) of 0.647. The topology of the strict consensus tree (Supplementary Fig. 4) shows a well resolved clade Sauropoda with diplodocoids and macronarians showing distinct clustering within Neosauropoda, consistent with previous studies^7,13^. *Tharosaurus* is recovered as a dicraeosaurid flagellicaudatan, although the clade Dicraeosauridae is poorly resolved. In the 50% majority rule tree (Fig. 6), Dicraeosauridae is better resolved with *Tharosaurus* being a sister taxon to ((*Pilmatueia* + *Amargatitanis*) + (*Brachytrachelopan* + (*Dicraeosaurus* + *Amargasaurus*))).

## Discussion

### Phylogenetic implications

*Tharosaurus* shares five synapomorphies with Flagellicaudata [divided lateral pleuroceols on cervical centra (ch. 115); bifurcated presacral neural spines (ch. 376); lateroventral flanges on middle/posterior cervical centra (ch. 382); heart-shaped anterior caudal centra cross-section (389); pre-epipophysis on middle/posterior cervicals (ch. 393)] and one unambiguous synapomorphy supports its recovery as a dicraeosaurid [divided cprl in cervicals with the medial lamina connecting with the intraprezygapophyseal lamina (127)]. *Tharosaurus* also shares two synapomorphies with other diplodocoids [transversely concave ventral surface of cervical centra (ch. 112); quadrangular middle caudal centra (ch. 208)]. Furthermore, three autapomorphies characterize *Tharosaurus*—smooth and narrow prespinal lamina on non-bifid dorsal neural spine (ch 143), pleuroceol on anterior caudal (ch 194), ventral mid-line keel in middle/posterior cervicals bifurcated posteriorly but not meeting the lateroventral flanges (ch 380). Barring the last autapomorphy, the remaining are local, being also present in a few diplodocids.

To further test the position of *Tharosaurus* within Sauropodomorpha, two additional analyses were conducted (A1 and A2; Supplementary Notes 3 and 4) using an expanded version of the data matrix of Gallina et al.^7^ which comprises a spatiotemporally and phylogenetically diverse array of sauropodomorphs, thereby allowing *Tharosaurus* to be placed anywhere within Sauropodomorpha. Furthermore, in analysis A2, characters involving cervical spines were scored as ‘?’, as these are not preserved in *Tharosaurus*, although the neural arch morphology strongly suggests the presence of bifurcated spines. Both analyses produce the same results where *Tharosaurus* is recovered as a dicraeosaurid (Supplementary Figs. 5–8). Furthermore, the phylogenetic bracketing of *Tharosaurus* by *Bajadasaurus* and ((*Pilmatueia* + *Amargatitanis*) + (*Brachytrachelopan* + (*Dicraeosaurus* + *Amargasaurus*))) in the majority rule tree of analysis A2 (Fig. 8), supports our original inference suggesting bifurcated cervical neural spines in *Tharosaurus*, as the bracketing taxa also possess the same feature.

### Palaeobiogeography

Sauropods are considered to have originated in the Late Triassic/Early Jurassic^38,47^ but the origin and radiation of Neosauropoda and its major clades (Diplodocoidea and Macronaria) are still amongst the most contentious issues^48^. Non-neosauropods were restricted to eastern Gondwana (India and Zimbabwe) and parts of Laurasia (Thailand, Germany and China) during Late Triassic–Early Jurassic, suggesting possible physiological constraints to their dispersal to the Americas and Australia^47^, although sampling biases cannot be ruled out. Neosauropods possibly appeared and radiated during the late Early/ early Middle Jurassic, with Asia and North–South America being some of the areas occupied by their most recent common ancestors (MRCAs)^13,49,50^. Palaeogeographic reconstructions support this hypothesis since Gondwana and Laurasia remained united for much of the early Mesozoic as the supercontinent Pangaea^51^. Although the Tethys Ocean was a barrier between Europe + Asia and Gondwana, land connections between North America and South America + Africa during the Triassic and Early Jurassic would have allowed sauropod dispersal^51,52^. Similarly, dispersal between North America and Europe^53^ possibly occurred during the Middle Jurassic, although these continents were likely separated by a narrow epicontinental sea (the Viking Corridor) during the Early Jurassic^54^. However, by the early Bajocian (~ 175 Ma), sea-floor spreading started in the western Tethys, the Central Atlantic region and the Gulf of Mexico^55–57^, and global transgressions flooded most continental shelves during the Middle and Late Jurassic, separating North America from South America and Africa^56,57^. Consequently, major neosauropod clades in these Laurasian and Gondwanan continents must have originated and radiated, prior to the Bajocian rifting^57^.

Until recently, the East Asian Jurassic dinosaurs were regarded as endemic fauna, characterized by mamenchisaurids and tetanurans^13,58^. Dispersal of neosauropods into East Asia apparently occurred only during the Early Cretaceous with the appearance of titanosauriforms, whereas the absence of diplodocoids was thought to be a result of reduced diversity and geographical range due to an end-Jurassic extinction event^13^. Faunal differences between East Asia and the rest of Laurasia were explained by the presence of an epicontinental seaway isolating Central and East Asia from the rest of Laurasia during the Middle and Late Jurassic^49,59^. Nonetheless, discoveries of the Chinese Middle and Late Jurassic neosauropods (*Dashanpusaurus*, *Lingwulong*, *Bellusaurus*) tend to weaken the isolation hypothesis^13,50^ and support a circum-Pangaean distribution of the major neosauropod clades by Middle Jurassic^13,50^. The age of the Chinese macronarian *Dashanpusaurus* is controversial but possibly Bajocian^50^, whereas the age and stratigraphic horizon in which the dicraeosaurid *Lingwulong* was found has recently been revised from the late Toarcian–Bajocian Yanan Formation to the Bathonian-Callovian Zhiluo Formation^50,60^. In their palaeobiogeographic analysis of sauropods, Ren et al.^50^ (p. 6, Fig. 3) assigned a Callovian age to *Lingwulong*. Thus, the discovery of *Tharosaurus* assumes considerable significance owing to its older, Early–Middle Bathonian age.

**Figure 7:**
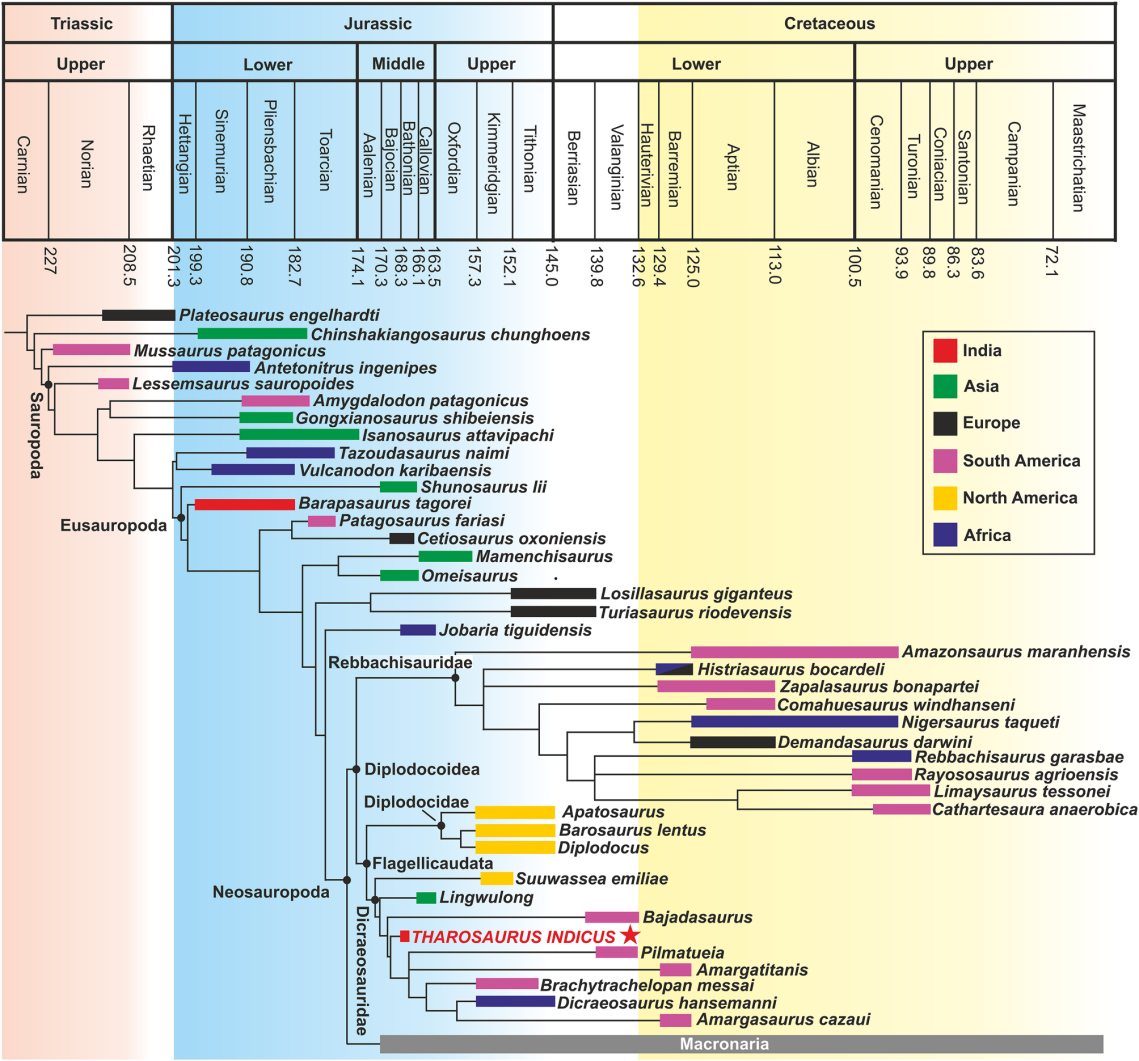
Time-calibrated phylogenetic tree, based on the 50% majority-rule tree of Supplementary Fig. 6. Macronarians have been combined into a single lineage to enhance clarity. Red star indicates position of *Tharosaurus indicus*.

**Figure 8:**
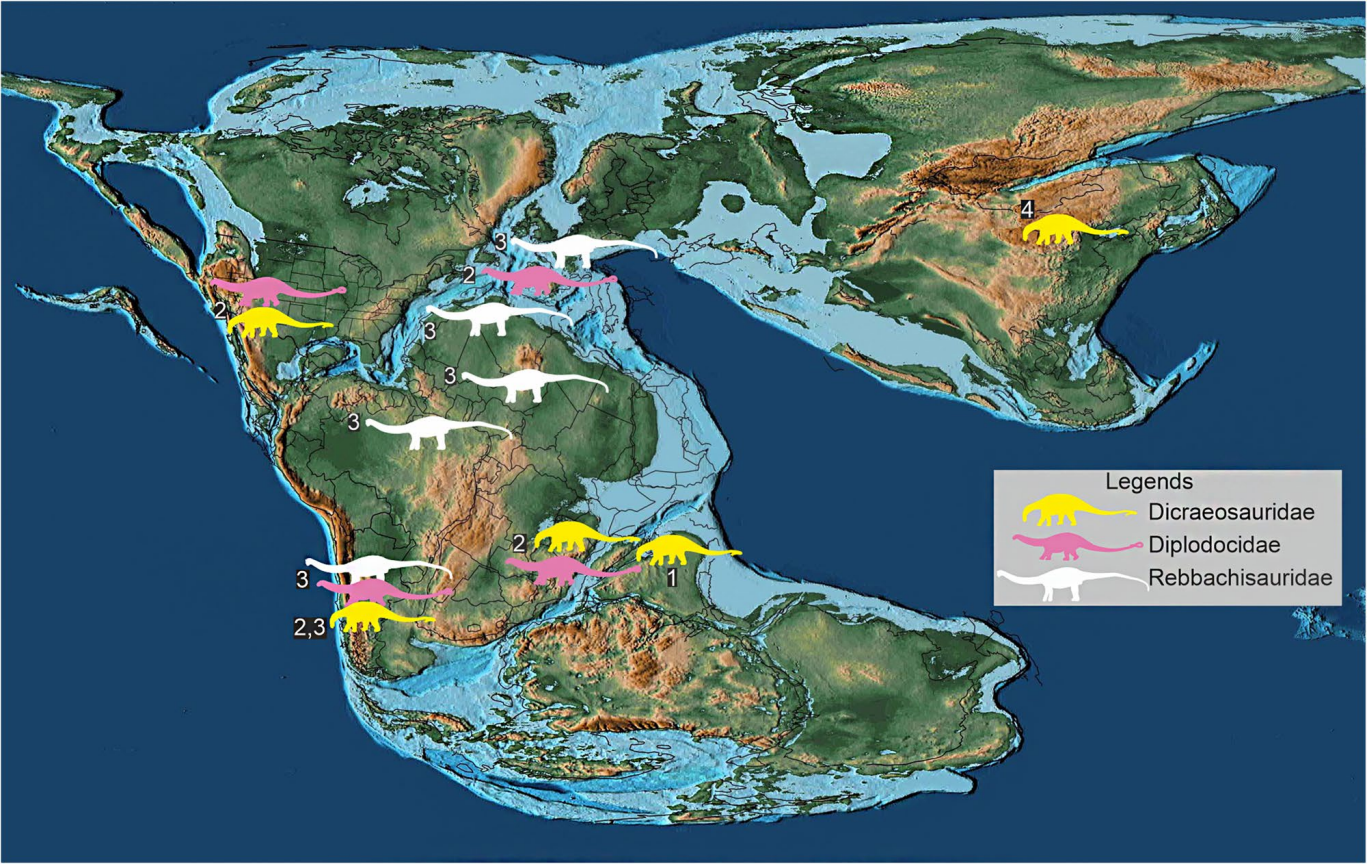
Palaeogeographic distribution of diplodocoids with taxa of different ages plotted together in a simplified Middle Jurassic (170 Ma) map to show their spatio-temporal distribution across Pangea. Silhouettes indicate the type of diplodocoid and fossil occurrences. Numbers adjoining sauropod silhouettes indicate age of the fossils as follows: 1—Middle Jurassic (early–middle Bathonian); 2—Late Jurassic; 3—Cretaceous; 4—Middle Jurassic (Callovian). Palaeogeographic map after Scotese^67^ and sourced from https://www.earthbyte.org/paleomap-paleoatlas-for-gplates/ [This work is licensed under the Creative Commons Attribution 4.0 International License. http://creativecommons.org/licenses/by/4.0/]. Source of information on sauropod distribution from the Paleobiology database (https://www.paleobiodb.org/) and Ren et al.^50^.

To assess the palaeobiogeographic significance of *Tharosaurus*, a time-calibrated phylogenetic tree was constructed (Figs. 7, 8). The resultant topology is consistent with the current consensus on rebbachisaurid origins^29,61,62^ as it suggests a largely Gondwanan origin since most of the taxa are from South America and Africa. Although the oldest rebbachisaurid in this study, *Histriasaurus*, is from Croatia, it is regarded as a Gondwanan taxon because Croatia was part of the Adriatic-Dinaric Carbonate Platform during the Early Cretaceous, sharing biotic affinities with Africa^61,63^. Based on the Late Jurassic *Maraapunisaurus*^64^, a North American origin of rebbachisaurids and their dispersal into South America through Europe and Africa during the latest Jurassic–earliest Cretaceous has been suggested. However, the rebbachisaurid affinity of this sauropod is questionable^29^. Early Cretaceous rebbachisaurids from England including *Xenoposeidon*, claimed to be the oldest member of this clade^29,62^, also add uncertainty to the Gondwanan origin of Rebbachisauridae. The lower-level phylogenetic affinity of these sauropods remains unresolved and further work is necessary to better understand rebbachisaurid origins. Our study recovers a long rebbachisaurid ghost lineage extending into the Middle Jurassic, corroborating recent work on the timing of origin and dispersal of this clade^13,50,65^. This work also suggests that differentiation within Rebbachisauridae started during the Late Jurassic, consistent with previous studies^53,64^.

The diplodocids included in our time-calibrated tree are from the Upper Jurassic horizons of North America (Figs. 7, 8) and suggest a North American origin for this clade. Current consensus on diplodocid taxonomy supports this inference since most of the other valid taxa (*Brontosaurus*, *Galeamopus*, *Supersaurus* and *Amphicoelias*) are also from North America^29,30,37^. Furthermore, the tree topology supports a pre-Bajocian divergence and dispersal of diplodocids from dicraeosaurids, consistent with most previous studies on flagellicaudatan dispersal and palaeobiogeography^29,57,65^. Diplodocids were, however, not restricted to North America, but are also known from the Late Jurassic of Europe (*Dinheirosaurus*^66^) and Africa (*Tornieria*^56^), and the Early Cretaceous of South America (*Leinkupal*^57^).

The clade Dicraeosauridae is represented by nine taxa ranging from Middle Jurassic to Early Cretaceous (Figs. 7, 8). The tree topology shows a late-diverging clade with a preponderance of South American dicraeosaurids (*Pilmatueia*, *Amargatitanis*, *Brachytrachelopan* and *Amargasaurus*) along with the African *Dicraeosaurus*, and argues for a Gondwanan origin for this clade. However, recovery of the Late Jurassic *Suuwassea* as the earliest-diverging dicraeosaurid raises the possibility of a North American origin for Dicraeosauridae and its subsequent migrations to South America and Africa, as initially proposed by Whitlock and Wilson Mantilla^29^. Recent work on flagellicaudatan palaeobiogeography based on the dicraeosaurid *Lingwulong* identified Asia + South America as some of the areas for MRCAs of Dicraeosauridae^13^.

Although later-diverging than *Suuwassea* and *Lingwulong,* the discovery of *Tharosaurus* in a much older Middle Jurassic horizon calls into question the above two hypotheses. The early–middle Bathonian age of *Tharosaurus* (see Supplementary Note 1) makes it the oldest diplodocoid globally. Even if *Lingwulong* is reinstated to its original estimated age of 174 Ma (sensu Xu et al*.*^13^), *Tharosaurus* remains the oldest Gondwanan diplodocoid as the African and South American diplodocoids appear from the Kimmeridgian (157 Ma)^13,64^ (Fig. 7). The sister taxon relationship between the new Indian taxon and the later-diverging African and South American dicraeosaurids indicates ease of faunal exchanges between India and western Gondwana. Based on the age of *Tharosaurus* and its phylogenetic position near the base of the clade Dicraeosauridae (Fig. 7), India (or a geographically proximate region of eastern Gondwana) is hypothesized here as a potential centre for the radiation and perhaps origin of dicraeosaurids/diplodocoids. Current palaeogeographic reconstructions^67^ lend support to this hypothesis since plausible dispersal routes from India to western Gondwana–Laurasia, through Madagascar, still remained in place during the Middle Jurassic. Furthermore, the estimated ancestral ranges depicted by the time-calibrated tree show the origin of Neosauropoda and its major clades—Macronaria + Diplodocoidea—to straddle the late Early Jurassic–early Middle Jurassic interval, consistent with recent studies on neosauropod origins^13,50,65^.

Our proposal favouring possible diplodocoid radiation/origin in India still needs to be reconciled with the phylogenetically more basal Asian taxon *Lingwulong*, the only other Middle Jurassic dicraeosaurid apart from *Tharosaurs*. An explanation for these two geographically disparate occurrences is perhaps a circum-Pangaean dispersal event. A direct dispersal to or from Asia is precluded by the Tethys Ocean which acted as a major barrier to terrestrial fauna during the Mesozoic. Furthermore, there is little support for the introduction of diplodocoids into India from Asia via North America and western Gondwana, since *Tharosaurus* is geologically older and phylogenetically early-branching relative to nearly all African and South American diplodocoids (Figs. 7, 8). The only exception is the South American *Bajadasaurus*, which is phylogenetically bracketed by *Suuwassea* + *Lingwulong* and *Tharosaurus*, but known from a much younger stratigraphic horizon (Fig. 7). The phylogenetic position of *Bajadasaurus* may possibly be explained by a radiation event from North America or from India predating *Tharosaurus*. However, additional sampling leading to greater anatomical coverage of *Tharosaurus* in the future may change its phylogenetic position. In any case, the long ghost lineage leading to *Bajadasaurus* presents the possibility of finding earlier-diverging taxa in western Gondwana.

Thus, a more plausible hypothesis, based on the older stratigraphic age of *Tharosaurus*, is that migrations from India to Asia could have taken place through western Gondwana and North America via Europe (Fig. 8). However, *Lingwulong* still poses a biogeographic problem, being older than all western Gondwanan and Laurasian diplodocoids. The biogeographic puzzle arising from the two Middle Jurassic dicraeosaurids in India and China indicates a pre-Middle Jurassic origin of neosauropods followed by widespread dispersal, as inferred in the present work and previous studies^13,50,65^. The lack of corroborative fossils representing temporally and phylogenetically intermediate taxa appears to be a consequence of sampling and geological biases^13^, and points to the need for more rigorous collecting efforts in these regions.

Despite the fragmentary material currently available and the possibility that the taxonomic attribution within Flagellicaudata may potentially change with the recovery of additional material/character sampling, *Tharosaurus* remains the oldest known diplodocoid. Together with the putative Bajocian camarasauromorph from Kutch^17^, the discovery of *Tharosaurus* makes India a major centre for not only diplodocoid but neosauropod radiation. While previous authors^13,50^ have considered Asia and the Americas as regions inhabited by the MRCAs of these clades, this study highlights the importance of pre-Bathonian Indian sauropodomorph record in tracing the origins of Neosauropoda. This suggestion is strongly supported by the well-known early-diverging non-neosauropods, *Barapasaurus* and *Kotasaurus*, from the Early Jurassic (Sinemurian–Pliensbachian) Kota Formation of India^18,19^. To conclude, the discovery of *Tharosaurus* emphasizes the need for increased sampling of Bathonian and older Jurassic horizons of India in search of ancestral taxa intermediate between the neosauropods and *Barapasaurus*-like eusauropods.

## Methods

### Osteological description

The osteological description of the skeletal specimens was carried out following the nomenclature of Bandyopadhyay et al.^2^, Coria et al.^8^ and Xu et al.^13^. Different parameters of the fossil specimens were measured (Supplementary Fig. 2) using Mitutoyo digital callipers with a precision of 0.01 mm. Explanatory line drawings are used wherever necessary. The terminology for vertebral laminae and fossae follows Wilson^25^ and Wilson et al.^32^.

### Phylogenetic analysis

The phylogenetic affinity of *Tharosaurus* was determined through an analysis based on a combination of characters from the datasets of Xu et al.^13^ and Gallina et al.^7^ with 19 additional characters from Tschopp et al.^26^ (Supplementary Data 1). The taxa-character matrix included 37 taxa and 394 characters. The phylogenetic analysis was performed in TNT version 1.6^68^ where the software memory was set to retain 10,000 trees with a display buffer of 10 Mb (sensu Coria et al.^8^). Following Gallina et al.^7^ the Traditional Search option was used to analyse the dataset. The constraints for the analysis included 5000 replications of Wagner trees, where the swapping algorithm was bisection reconnection with 10 trees saved per replication. To determine the robustness of the nodes, Bremer support values were calculated using the script bremer.run where only trees suboptimal by 20 steps were retained. The outgroup taxon in this analysis was *Shunosaurus lii*.

### Supplementary Information

Supplementary Information 1.

Supplementary Information 2.

Supplementary Information 3.

Supplementary Information 4.

## Data Availability

All data associated with the manuscript are provided in the supplementary file.

## Abbreviations

ANS: Academy of Natural Sciences, Philadelphia, USA
MACN: Museo Argentino de Ciencias Naturales, Buenos Aires, Argentina
YPM: Yale Peabody Museum, New Haven, USA

## Code availability

**Nomenclatural acts.** This published work and the nomenclatural acts it contains have been registered in ZooBank, the proposed online registration system for the International Code of Zoological Nomenclature (ICZN). The LSIDs for this publication are urn:lsid:zoobank.org:act:EB6CB405-6FD6-4EDA-9E49-ED08105AFBD8 (*Tharosaurus*) and urn:lsid:zoobank.org:act:C43C0866-88BB-475B-9439-86C8837AB833 (*T. indicus*).
