# Author Correction: Fossils of the oldest diplodocoid dinosaur suggest India was a major centre for neosauropod radiation

**DOI:** 10.1038/s41598-023-40641-4

**Published:** 2023-08-21

**Authors:** Sunil Bajpai, Debajit Datta, Pragya Pandey, Triparna Ghosh, Krishna Kumar, Debasish Bhattacharya

**Affiliations:** 1 Department of Earth Sciences, Indian Institute of Technology, Roorkee, Uttarakhand 247667 India; 2 Geological Survey of India, Raipur, Chhattisgarh 492010 India; 3 Geological Survey of India, Jaipur, Rajasthan 302004 India; 4 Central Head Quarters, Geological Survey of India, Kolkata, West Bengal 700091 India

Correction to: *Scientific Reports* 10.1038/s41598-023-39759-2, published online 04 August 2023

The original version of this Article contained an error in the Results section, under the subheading ‘Systematic palaeontology’, where the expanded form of the specimen number “RWR-241(A–K)” was missing. Consequently,

“*Holotype.* RWR-241 A–K;”

now reads:

“*Holotype.* RWR-241 A–K (Palaeontology Division, Geological Survey of India, Western Region, Jaipur, Rajasthan, India);”

The original Article has been corrected.

