# Supplementary material for: Fossils of the oldest diplodocoid dinosaur suggest India was a major centre for neosauropod radiation
Source: Sci Rep. 2023 Aug 4;13:12680. doi: 10.1038/s41598-023-39759-2 (PMC10403599; doi:10.1038/s41598-023-39759-2)
Supplement: Supplementary file 3 — Supplementary Information 3. [file 41598_2023_39759_MOESM3_ESM.pdf]

Supplementary Information for

Fossils of the oldest diplodocoid dinosaur suggest India was a major centre for neosauropod radiation

Sunil Bajpai[1,*], Debajit Datta[1,*], Pragya Pandey[2], Triparna Ghosh[1,3], Krishna Kumar[3] & Debasish Bhattacharya[4]

[1]Department of Earth Sciences, Indian Institute of Technology, Roorkee, Uttarakhand 247667, India; debajitdatta.pd@es.iitr.ac.in; debajitdatta9@gmail.com; sunil.bajpai@es.iitr.ac.in

[2]Geological Survey of India, Raipur, Chhattisgarh 492010, India; pragya.pandey@gsi.gov.in

[3]Geological Survey of India, Jaipur, Rajasthan 302004, India; krishna.kumar@gsi.gov.in; triparna.ghosh@gsi.gov.in

[4]Central Head Quarters, Geological Survey of India, Kolkata, West Bengal 700091, India; debasish.bhattacharya@gsi.gov.in

Supplementary Information

1. Supplementary Note 1: Geological setting of the Jaisalmer Basin
2. Supplementary Note 2: List of characters used in the phylogenetic analysis
3. Supplementary Note 3: Phylogenetic analysis A1
4. Supplementary Note 4: Phylogenetic analysis A2
5. Supplementary Table 1: List of specimens for the holotype of *Tharosaurus indicus*
6. Supplementary Table 2: Measurements of skeletal specimens
7. Supplementary figures
8. Supplementary references

1. Supplementary Note 1: Geological Setting

The Jaisalmer Basin is a pericratonic shelf basin in the north-western margin of the Indian craton[1–2]. It preserves a thick sequence of Early Jurassic–Middle Eocene marine siliciclastic carbonate deposits that rest unconformably on the Precambrian basement[2–3]. The Jurassic sedimentary units are preserved in the Lathi, Jaisalmer, Baisakhi and Bhadasar formations, in succeeding order, and the most fossiliferous among these is the Jaisalmer Formation (Supplementary Fig. 1A). The latter ranges from Late Bajocian–Oxfordian and is characterized by cross-bedded sandstones, siltstones, and limestones[3]. Six members constitute the Jaisalmer Formation and include, in chronological order, the Hamira, Joyan, Fort, Badabag, Kuldhar, and Jajiya, member[3–4]. The study area is in the Fort Member, situated along the Jaisalmer-Kanod Road, and about 3 km SE of Jethwai village (N 27°00′36.7”; E 70°56′56.6”; Supplementary Fig. 1A–C). The Fort Member consists of shale at the base followed by an alteration of the cross-bedded sandstone and siltstone which is succeeded by argillaceous marl and limestone (Supplementary Fig. 1B). Furthermore, associated fauna from this member includes fish (hybodont sharks, pycnodonts), crocodylians and a putative theropod, and invertebrates such as bivalves, brachiopods, and ammonites[1–3,5].

An Early–Middle Bathonian age has been assigned to the Fort Member as it stratigraphically rests above the Late Bajocian coral (*Isastrea bernardiana*) bearing Joyan member and underlies the Badabag Member from where Late Bathonian ammonites (*Perisphinctes congener*, *Macrocephalites madagascariensis, M. triangularis, M. formosus* and *Sivajiceras congener*) are reported [1–2]. This Early–Middle Bathonian age of the Fort Member is further supported by interbasinal corelation of marker beds[6].

.

2. Supplementary Note 2: Characters used in the phylogenetic analysis. The citations are abbreviated as follows: X, Xu *et al.*[7]; G, Gallina *et al.*[8]; T, Tshcopp *et al.*[9], whereas the numerals indicate the character number of each citation.

1. Posterolateral processes of premaxilla and lateral processes of maxilla, shape: without midline contact (0); with midline contact forming marked narial depression, subnarial foramen not visible laterally (1). (X1, G1)
2. Premaxillary anterior margin shape: without step (0); with marked step but short step (1); with marked and long step (2). (X2, G2)
3. Premaxila, ascending process shape in lateral view: convex (0); concave, with a large dorsal projection (1); sub-rectilinear and directed posterodorsally (2). (X3, G3)
4. Premaxilla, external surface: without anteroventrally orientated vascular grooves originating from an opening in the maxillary contact (0); vascular grooves present (1). (X4, G4)
5. Maxillary border of external naris, length: short, making up much less than one-fourth narial perimeter (0); long, making up more than one third narial perimeter (1). (X5, G5)
6. Maxilla, foramen anterior to the preantorbital fenestra: absent (0); present (1). (X6, G6)
7. Preanteorbital fenestra: absent (0); present, being wide and laterally opened (1). (X7, G7)
8. Subnarial foramen and exterior maxillary foramen, position: well distanced from one another (0); separated by narrow bony isthmus (1). (X8, G8)
9. Antorobital fenestra: much shorter than orbital maximum diameter, less than 85% of orbit (0); subequal to orbital maximum diameter, greater than 85% orbit (1). (X9, G9)

10. Antorbital fenestra, shape of dorsal margin: straight or convex (0); concave (1).(X10, G10)
11. Antorbital fossa: present (0); absent (1). (X11, G11)
12. External nares position: terminal (0); retracted to level of orbit (1); retracted to a position between orbits (2). (X12, G12)
13. External nares, maximum diameter: shorter (0); or longer than orbital maximum diameter (1). (X13, G13)
14. Orbital ventral margin, anteroposterior length: broad, with subcircular orbital margin (0); reduced, with acute orbital margin (1). (X14, G14)
15. Lacrimal, anterior process: present (0); absent (1). (X15, G15)
16. Jugal contribution to the ventral border of the skull: present and long (0); absent or veryreduced (1). (X16, G16)
17. Quadratojugal-Maxilla contact: absent or small (0); broad (1). (X17, G17)
18. Jugal-ectopterygoid contact: present (0); absent (1). (X18, G18)
19. Jugal, contribution to antorbital fenestra: absent (0); present, but very reduced (1); present and large, bordering approximately one-third of its perimeter (2). (X19, G19)
20. Quadratojugal, position of anterior terminus: posterior to middle of orbit (0); anterior margin of orbit or beyond (1). (X20, G,20)
21. Quadratojugal, anterior process length: short, anterior process shorter than dorsal process (0); long, anterior process more than twice as long as dorsal process (1). (X21, G21)
22. Quadratojugal, angle between anterior and dorsal processes: less than or equal to 90°, sothat the quadrate shaft is directed dorsally (0); greater than 90°, approaching 130°, so that the quadrate shaft slants posterodorsally (1). (X22, G,22)

23. Ventral edge of anterior surface of the quadratojugal: straight, not expanded ventrally(0); slightly expanded ventrally, forming a small bulge, the height of which is less thantwice the ramus height (1); well expanded ventrally, forming a notorious bulge, the height of which is twice or more the minimum height of the ramus (2). (X23, G23)
24. Squamosal contribution to the supratemporal fenestra: present, the squamosal is well visible in dorsal view (0); reduced or absent (1). (X24, G24)
25. Squamosal-quadratojugal contact: present (0); absent (1). (X25, G25)
26. Squamosal, posteroventral margin: smooth (0); "with prominent, ventrally directed ‘prong’ (1). (X26, G26)
27. Prefrontal posterior process size: small, not projecting far posterior of frontal-nasal suture (0); elongate, approaching parietal (1). (X27, G27)
28. Prefrontal, posterior process shape: flat (0); hooked (1). (X28, G28)
29. Prefrontal, anterior process: absent (0); present (1). (X29, G29)
30. Prefrontal-Frontal contact width: large, equal or longer that the anteroposterior length ofthe prefrontal (0); narrow, less than half the anteroposterior length of the prefrontal (1). (X30, G30)
31. Postorbital, ventral process shape: transversely narrow (0); broader transversely than anteroposteriorly (1). (X31, G31)
32. Postorbital, posterior process: present (0); absent (1). (X32, G32)
33. Postorbital, posterior margin articulating with the squamosal: with tapering posterior process (0); with a deep posterior process (1). (X33, G33)
34. Frontal contribution to supratemporal fossa: present (0); absent (1). (X34, G34)
35. Frontals, midline contact (symphysis): sutured (0); or fused in adult individuals (1). (X35, G35)

36. Frontal, anteroposterior length: approximately twice (0); or less than minimum transverse breadth (1). (X36, G36)

37. Frontal-nasal suture, shape: flat or slightly bowed anteriorly (0); V-shaped, pointing posteriorly (1). (X37, G37)

38. Frontals, dorsal surface: without paired grooves facing anterodorsally (0); grooves present, extend on to nasal (1). (X38, G38)

39. Frontal, contribution to dorsal margin of orbit: contribution to dorsal margin of orbit: less than 1.5 times the contribution of prefrontal (0); at least 1.5 times the contributionof prefrontal (1). (X39, G39)

40. Parietal occipital process, dorsoventral height: short, less than the diameter of the foramen magnum (0); deep, nearly twice the diameter of the foramen magnum (1). (X40, G40)

41. Parietal, contribution to post-temporal fenestra: present (0); absent (1). (X41, G41)

42. Parietal, distance separating supratemporal fenestrae: less than the long axis of supratemporal fenestra, 0.8 or less (0); almost the same as the long axis of supratemporal fenestra 0.8–1.2 (1); much larger than the long axis of supratemporal fenestra; more than 1.2 (2). (X42, G42)

43. Postparietal foramen: absent (0); present (1). (X43, G43)

44. Paroccipital process distal terminus: straight, slightly expanded surface (0); rounded, tongue-like process (1). (X44, G44)

45. Supratemporal fenestra: present (0); absent (1). (X45, G45)

46. Supratemporal fenestra, long axis orientation: anteroposterior (0); transverse (1). (X46, G46)

47. Supratemporal fenestra, maximum diameter: much longer than (0); or subequal to that of foramen magnum (1). (X47, G47)

48. Supratemporal region, anteroposterior length: temporal bar longer (0); or shorter anteroposteriorly than transversely (1). (X48, G48)

49. Supratemporal fossa, lateral exposure: not visible laterally, obscured by temporal bar (0); visible laterally, temporal bar shifted ventrally (1). (X49, G49)

50. Supraoccipital, sagital nuchal crest: broad, weakly developed (0); narrow, sharp and distinct (1). (X50, G50)

51. Laterotemporal fenestra, anterior extension: posterior to orbit (0); ventral to orbit (1). (X51, G51)

52. Quadrate fossa: absent (0); present (1). (X52, G52)

53. Quadrate fossa, depth: shallow (0); deeply invaginated (1). (X53, G53)

54. Quadrate fossa, orientation: posterior (0); posterolateral (1). (X54, G54)

55. Quadrate, articular surface shape: quadrangular in ventral view, oriented transversely (0); roughly triangular in shape or thin, crescent-shaped surface with anteriorly directed medial process (1). (X55, G55)

56. Quadrate, articular surface shape: quadrangular in ventral view, oriented transversely or roughly triangular in shape (0); thin, crescent-shaped surface with anteriorly directed medial process (1). (X56, G56)

57. Palatobasal contact, shape: pterygoid with small facet (0); dorsomedially orientated hook (1); or rocker-like surface for basipterygoid articulation (2). (X57, G57)

58. Pterygoid, transverse flange (i.e. ectopterygoid process) position: posterior of orbit (0);between orbit and antorbital fenestra (1); anterior to antorbital fenestra (2). (X58, G58)

59. Pterygoid, quadrate flange size: large, palatobasal and quadrate articulations well separated (0); small, palatobasal and quadrate articulations approach (1). (X59, G59)

60. Pterygoid, palatine ramus shape: straight, at level of dorsal margin of quadrate ramus (0); stepped, raised above level of quadrate ramus (1). (X60, G60)
61. Pterygoid, sutural contact with ectopterygoid: broad, along the medial or lateral surface (0); narrow, restricted to the anterior tip of the ectopterygoid (1). (X61, G61)
62. Palatine, lateral ramus shape: plate-shaped (long maxillary contact) (0); rod-shaped (narrow maxillary contact) (1). (X62, G62)
63. Epipterygoid: present (0); absent (1). (X63, G63)
64. Vomer, anterior articulation: maxilla (0); premaxilla (1). (X64, G64)
65. Supraoccipital, height: twice subequal to (0); or less than height of foramen magnum (1). (X65, G65)
66. Paroccipital process, ventral non-articular process: absent (0); present (1). (X66, G66)
67. Crista prootica, size: rudimentary (0); expanded laterally into dorsolateral process (1). (X67, G67)
68. Basipterygoid processes, length: short, approximately twice (0); or elongate, at least four times basal diameter (1). (X68, G68)
69. Basipterygoid processes, angle of divergence: approximately 45° (0); less than 30° (1). (X69, G69)
70. Basal tubera, anteroposterior depth: approximately half dorsoventral height (0); sheet-like, 20% dorsoventral height (1). (X70, G70)
71. Basal tubera, breadth: much broader than (0); or narrower than occipital condyle (1). (X71, G71)
72. Basal tubera: distinct from basipterygoid (0); reduced to slight swelling on ventral surface of basipterygoid (1). (X72, G72)
73. Basal tubera, shape of posterior face: convex (0); slightly concave (1). (X73, G73)

74. Basioccipital depression between foramen magnum and basal tubera: absent (0); present(1). (X74, G74)

75. Basisphenoid/basipterygoid recess: present (0); absent (1). (X75, G75)

76. Basisphenoid/quadrate contact: absent (0); present (1). (X76, G76)

77. Basisphenoid, sagital ridge between basipterygoid processes: absent (0); present (1). (X77, G77)

78. Basipterygoid processes, orientation: perpendicular to (0); or angled approximately 45° to skull roof (1). (X78, G78)

79. Basipterygoid, area between the basipterygoid processes and parasphenoid rostrum: is a mildly concave subtriangular region (0); forms a deep slot-like cavity that passes posteriorly between the bases of the basipterygoid processes (1). (X79, G79)

80. Occipital region of skull, shape: anteroposteriorly deep, paroccipital processes oriented posterolaterally (0); flat, paroccipital processes oriented transversely (1). (X80, G80)

81. Dentary, depth of anterior end of ramus: slightly less than that of dentary at midlength(0); 150% minimum depth (1). (X81, G81)

82. Dentary, anteroventral margin shape: gently rounded (0); sharply projecting triangular process (1). (X82, G82)

83. Dentary symphysis, orientation: angled 15° or more anteriorly to (0); or perpendicular to axis of jaw ramus (1). (X83, G83)

84. Dentary, cross-sectional shape of symphysis: oblong or rectangular (0); subtriangular, tapering sharply towards ventral extreme (1); subcircular (2). (X84, G84)

85. Dentary, tuberosity on labial surface near symphysis: absent (0); present (1). (X85, G85)

86. Mandible, coronoid eminence: strongly expressed, clearly rising above plane of dentigerous portion (0); absent (1). (X86, G86)

87. External mandibular fenestra: present (0); absent (1). (X87, G87)

88. Surangular depth: less than twice (0); or more than two and one-half times maximum depth of the angular (1). (X88, G88)

89. Surangular ridge separating adductor and articular fossae: absent (0); present (1). (X89, G89)

90. Adductor fossa, medial wall depth: shallow (0); deep, prearticular expanded dorsoventrally (1). (X90, G90)

91. Splenial posterior process, position: overlapping angular (0); separating anterior portions of prearticular and angular (1). (X91, G91)

92. Splenial posterodorsal process: present, approaching margin of adductor chamber (0); absent (1). (X92, G92)

93. Coronoid, size: extending to dorsal margin of jaw (0); reduced, not extending dorsal tosplenial (1); absent (2). (X93, G93)

94. Tooth rows, shape of anterior portions: narrowly arched, anterior portion of tooth rowsV-shaped (0); broadly arched, anterior portion of tooth rows U-shaped (1); rectangular, tooth-bearing portion of jaw perpendicular to jaw rami (2). (X94, G94)

95. Tooth rows, length: extending to orbit (0); restricted anterior to orbit (1); restricted anterior to antorbital fenestra (2); restricted anterior to subnarial foramen (3). (X95, G95)

96. Dentary teeth, number: greater than 20 (0); 10–17 (1); 9 or fewer (2). (X96, G96)

97. Replacement teeth per alveolus, number: two or fewer (0); more than four (1). (X97, G97)

98. Lateral plate: absent (0); present (1). (X98, G98)

99. Teeth, orientation: perpendicular (0); or oriented anteriorly relative to jaw margin (1). (X99, G99)

100. Tooth crowns, orientation: aligned along jaw axis, crowns do not overlap (0); aligned slightly anterolingually, tooth crowns overlap (1). (X100, G100)

101. Crown-to-crown occlusion: absent (0); present (1). (X101, G101)

102. V-shaped wear facets: present (0); absent (1). (X102, G102)

103. Tooth crowns, cross-sectional shape at mid-crown: elliptical (0); D-shaped (1); sub-cylindrical (2); cylindrical (3). (X103, G103)

104. Enamel surface texture: smooth (0); wrinkled (1). (X104, G104)

105. Thickness of enamel asymmetric labiolingually: absent (0); present (1). (X105, G105)

106. Marginal tooth denticles: present (0); absent on posterior edge (1); absent on both anterior and posterior edges (2). (X106, G106)

107. Teeth, longitudinal grooves on lingual aspect: absent (0); present (1). (X107, G107)

108. SI values for tooth crowns: less than 3.0 (0); 3.0–4.0 (1); 4.0–5.0 (2); more than 5.0 (3). (X108, G108)

109. Cervical vertebrae, number: 10 or fewer (0); 12 (1); 13–14 (2); 15 (3); 16 or more (4). (X109, G109)

110. Atlas, intercentrum occipital facet shape: rectangular in lateral view, length of dorsal aspect subequal to that of ventral aspect (0); expanded anteroventrally in lateral view, anteroposterior length of dorsal aspect shorter than that of ventral aspect (1). (X110, G110)

111. Cervical centra, articulations amphicoelous (0); opisthocoelous (1). (X111, G111)

112. Cervical centra, ventral surface: is flat or slightly convex transversely (0); transversely concave (1). (X112, G112)

113. Cervical centra, midline keels on ventral surface: prominent and plate-like (0); reduced to low ridges or absent (1). (X113, G113)

114. Cervical centra, pleurocoels: absent (0); present with well defined anterior, dorsal, and ventral edges, but not the posterior one (1); present, with well defined edges; present but very reduced in size (3). (X114, G114)

115. Cervical centra, pleurocoels: singles without division (0); with a well defined anterior excavation and a posterior smooth fossa (1); divided by a bone septum, resulting in an anterior and a posterior lateral excavation (2); divided in three or more lateral excavations, resulting in a complex morphology (3); with a well defined anterior excavation and a posterior smooth fossa. (X115, G115)

116. Cervical vertebrae, height divided width (measured in its posterior articular surface): higher than 1.1 (0), around 1 (1); between 0.9 and 0.7 (2); smaller than 0.7 (3). (X116, G116)

117. Cervical centra, small notch in the dorsal margin of the posterior articular surface: absent (0); present (1). (X117, G117)

118. Cervical vertebrae, neural arch lamination: well developed, with well marked laminaeand fossae (0); rudimentary, with diapophyseal laminae absents or very slightly marked (1). (X118, G118)

119. Cervical vertebrae with an accessory lamina, which runs from the postzygodiapophyseal lamina (PODL) up to the spinoprezygapophyseal lamina (SPRL): absent (0); present (1). (X119, G119)

120. Cervical centra, internal pneumaticity: absent (0); present with singles and wide cavities (1); present, with several small and complex internal cavities (2). (X120, G120)

121. Anterior cervical vertebrae, prespinal lamina: absent (0); present (1). (X121, G121)

122. Anterior cervical vertebrae, neural spine shape: single (0); bifid (1). (X122, G122)

123. Middle and posterior cervical vertebrae, prespinal lamina: absent (0); present (1). (X123, G123)

124. Middle cervical vertebrae, lateral fossae on the prezygapophysis process: absent (0); present (1). (X124, G124)

125. Middle cervical vertebrae, height of the neural arch: less than the height of the posterior articular surface (0); higher than the height of the posterior articular surface (1). (X125, G125)

126. Middle cervical centrum, anteroposterior length divided the height of the posterior articular surface: less than 4 (0); more than 4 (1). (X126, G126)

127. Middle and posterior cervical vertebrae, morphology of the centroprezygapophyseal lamina: single (0); dorsally divided, resulting in a lateral and medial lamina, being the medial lamina linked with the intraprezygapophyseal lamina and not with the prezygapophysis (1); divided, resulting in the presence of a "true" divided centroprezygapophyseal lamina, which is dorsally connected to the prezygapophysis (2). (X127, G127)

128. Middle and posterior cervical vertebrae, morphology of the centropostzygapophyseal lamina (CPOL): single (0); divided, with the medial part contacting the intrapostzygapophyseal lamina (1) (X128, G128)

129. Middle and posterior cervical vertebrae, articular surface of zygapophyses: flat (0); transversally convex (1). (X129, G129)

130. Posterior cervical vertebrae, lateral profile of the neural spine: displays steeply sloping cranial and caudal faces (0); displays steeply sloping cranial face and noticeably less steep caudal margin (1). (X130, G130)

131. Posterior cervical vertebrae, neural spine shape: without a great lateral expansion (0); laterally expanded, being equal or wider than the vertebral centrum (1) (X131, G131)

132. Posterior cervical and anterior dorsal vertebrae, neural spine shape: single (0); bifid (1). (X132, G132)

133. Posterior cervical and anterior dorsal bifid neural spines, median tubercle: absent (0); present (1). (X133, G133)

134. Number of dorsal vertebrae: 14 or more (0); 13 (1); 12 (2); 10 (3). (X134, G134)

135. Dorsal centra, pleurocoels: absent (0); present (1). (X135, G135)

136. Dorsal vertebrae, transverse processes: are directed laterally or slightly upwards (0); are directed strongly dorsolaterally (1). (X136, G136)

137. Dorsal vertebrae, distal end of the transverse process: curves smoothly in to the dorsal surface of the process (0); is set off from the dorsal surface, the latter having a distinct dorsally facing flattened area (1). (X137, G137)

138. Dorsal vertebrae, non bifid neural spines in anterior or posterior view: possess subparallel lateral margins (0); possess lateral margins which slightly diverge dorsally (1); possess lateral margins which strongly diverge dorsally (2). (X138, G138)

139. Dorsal centra, pneumatic structures: absent, dorsal centra with solid interna structure (0); present, dorsal centra with simple and big air-spaces (camerate) (1); present, dorsal centra with small and complex air-spaces (polycamerate) (2); present, dorsal centra with small and complex air spaces (semicamellate/camellate) (3). (X139, G139)

140. Anterior and middle dorsal neural spines, spinoprezygapophyseal lamina (SPRL): absent (0); present (1). (X140, G140)

141. Posterior dorsal neural spines, spinoprezygapophyseal lamina (SPRL): absent (0); present (1). (X141, G141)

142. Dorsal vertebrae, single not bifid neural spines, single prespinal lamina (PRSL): absent (0); present (1). (X142, G142)

143. Dorsal vertebrae, single not bifid neural spines, single prespinal lamina (PRSL): rough and wide, present in the dorsalmost part of the neural spine (0); rough and wide, extended through almost all the neural spine (1); smooth and narrow (2). (X143, G143)

144. Dorsal vertebrae with single neural spines, middle single fossa projected throughout the midline of the neural spine: present (0); absent (1). (X144, G144)

145. Dorsal vertebrae with single neural spines, middle single fossa, projected through the midline of the neural spine: relatively wide median simple fossa (0); a thin median simple fossa (1); extremely reduced median simple fossa (2). (X145, G145)

146. Anterior dorsal centra, articular face shape: amphicoelous (0); opisthocoelous (1). (X146, G146)

147. Anterior and middle dorsal centra, pleurocoels: have rounded caudal margins (0); have tapering, acute caudal margins (1). (X147, G147)

148. Middle dorsal neural arches in lateral view, anterior edge of the neural spine: project anteriorly to the diapophysis (0); converge with the diapophysis (1); project posteriorly to the diapophysis (2). (X148, G148)

149. Anterior and middle dorsal vertebrae, zygapophyseal articulation angle: horizontal or slightly posteroventrally oriented (0); posteroventraly oriented (around 30º) (1); strongly posteroventraly oriented (more than 40º) (2). (X149, G149)

150. Middle to posterior dorsal centra, ventral surface: convex transversely (0); flattened (1);is slightly concave, sometimes with one or two crests (2). (X150, G150)

151. Middle dorsal vertebrae, hyposphene-hypantrum system: present (0); absent (1). (X151, G151)

152. Posterior dorsal vertebrae, hyposphene-hypantrum system: present and well developed, usually with a rhomboid shape (0); present and weakly developed, mainly as a laminar articulation (1); absent or only present in posteriormost dorsal vertebrae

(2). (X152, G152)

153. Middle and posterior dorsal vertebrae, transverse processes length: short (0); long (projecting along 1.5 the articular surface width) (1). (X153, G153)

154. Mid and posterior dorsal vertebrae with a single lamina (the single TPOL) supportingthe hyposphene or postzygapophysis from below: absent (0); present (1). (X154, G154)

155. Middle and posterior dorsal vertebrae, neural canal in anterior view: entirely surroundedby the neural arch (0); enclosed in a deep fossa, enclosed laterally by pedicels (1). (X155, G155)

156. Middle and posterior dorsal vertebrae, neural spine height: approximately twice the centrum length (0); for times the centrum length (1). (X156, G156)

157. Middle and posterior dorsal neural spines orientation: vertical (0); slightly inclined, withan angle of around 70 degrees (1); strongly inclined, with an angle not bigger than 40 degrees (2). (X157, G157)

158. Middle and posterior dorsal neural arches, centropostzygapophyseal lamina (CPOL), shape: simple (0); divided (1). (X158, G158)

159. Middle and posterior dorsal neural arches, anterior centroparapophyseal lamina (ACPL): absent (0); present (1). (X159, G159)

160. Middle and posterior dorsal neural arches, prezygoparapophyseal lamina (PRPL): absent (0); present (1). (X160, G160)

161. Middle and posterior dorsal neural arches, posterior centroparapophyseal lamina (PCPL): absent (0); present (1). (X161, G161)

162. Middle and posterior dorsal centrum in transverse section (height: width ratio): subcircular (ratio, similar to 1 or a bit higher) (0); slightly dorsoventrally compressed (ratios between 0.8 and 1) (1); strongly compressed (ratios below 0.8) (2). (X162,

G162)

163. Middle and posterior dorsal vertebrae neural spine, triangular aliform processes: absent (0); present but do not project far laterally (not as far as caudal zygapophyses) (1); present and project far laterally (as far as caudal zygapophyses) (2). (X163, G163)

164. Middle and posterior dorsal vertebrae, spinodiapophyseal lamina (SPDL): absent (0); present (1). (X164, G164)

165. Middle and posterior dorsal vertebrae, accessory spinodiapophyseal lamina: absent (0); present (1). (X165, G165)

166. Dorsal vertebrae, spinodiapophyseal webbing: lamina follows curvature of neural spine in anterior view (0); lamina "festooned" from spine, dorsal margin does not closely follow shape of neural spine and diapophysis (1). (X166, G166)

167. Anterior dorsal vertebrae, spinopostzygapophyseal lamina (SPOL): absent (0); present(1). (X167, G167)

168. Middle and posterior dorsal neural spines, lateral spinopostzygapophyseal lamina (lSPOL): absent (0); present (1). (X168, G168)

169. Middle and posterior dorsal neural arches, spinodiapophyseal lamina (SPDL) and spinopostzygapophyseal lamina (lSPOL) contact: absent (0); present (1). (X169, G169)

170. Middle and posterior dorsal vertebrae, spinodiapophyseal (SPDL) and spinopostzygapophyseal lamina (lSPOL) contact: ventral, well separated from the triangular aliform process (0); dorsal, forms part of the triangular aliform process (1). (X170, G170)

171. Middle and posterior dorsal vertebrae, height of neural arch below the postzygapophyses (pedicel): less than height of centrum (0); subequal to or greater than height of centrum (1). (X171, G171)

172. Posterior Dorsal vertebrae, medial spinopostzygapophyseal lamina (mSPOL): absent (0); present and forms part of the median posterior lamina (1). (X172, G172)

173. Posterior dorsal vertebrae, transverse processes: lie posterior, or posterodorsal, to the parapophysis (0); lie vertically above the parapophysis (1). (X173, G173)

174. Posterior dorsal centra, articular face shape: amphicoelous (0); slightly opisthocoelous (1); opisthocoelous (2). (X174, G174)

175. Posterior dorsal vertebrae, neural spine: narrower transversely than anteroposteriorly (0); broader transversely than anteroposteriorly (1). (X175, G175)

176. Posterior dorsal vertebra, posterior centrodiapophyseal lamina (PCDL): has an unexpanded ventral tip (0); expands and may bifurcate toward its ventral tip (1). (X176, G176)

177. Cervical ribs, distal shafts of longest cervical ribs: are elongate and form overlapping bundles (0); are short and do not project beyond the caudal end of the centrum to whichthey are attached (1). (X177, G177)

178. Cervical ribs, angle between the capitulum and tuberculum: greater than 90°, so that the rib shaft lies close to the ventral edge of the centrum (0); less than 90°, so that the rib shaft lies below the ventral margin of the centrum (1). (X178, G178)

179. Dorsal ribs, proximal pneumatopores: absent (0); present (1). (X179, G179)

180. Anterior dorsal ribs, cross-sectional shape: subcircular (0); plank-like, anteroposterior breadth more than three times mediolateral breadth (1). (X180, G180)

181. Sacral vertebrae, number: 3 or fewer (0); 4 (1); 5 (2); 6 (3). (X181, G181)

182. Sacrum, sacricostal yoke: absent (0); present (1). (X182, G182)

183. Sacral vertebrae contributing to acetabulum: numbers 1–3 (0); numbers 2–4 (1). (X183, G183)

184. Sacral neural spines length: approximately twice length of centrum (0);

approximatelyfour times length of centrum (1). (X184, G184)

185. Sacral ribs, dorsoventral length: low, not projecting beyond dorsal margin of ilium (0);high extending beyond dorsal margin of ilium (1). (X185, G185)

186. Pleurocoels in the lateral surfaces of sacral centra: absent (0); present (1). (X186, G186)

187. Caudal vertebrae, number: 35 or fewer (0); 40 to 55 (1); increased to 70–80 (2). (X187, G187)

188. Caudal bone texture: solid (0); spongy, with large internal cells (1). (X188, G188)

189. Caudal transverse processes: persist through caudal 20 or more posteriorly (0); disappear by caudal 15 (1); disappear by caudal 10(2). (X189, G189)

190. First caudal centrum or last sacral vertebra, articular face shape: flat (0); procoelous (1); opisthocoelous (2); biconvex (3). (X190, G190)

191. First caudal neural arch, coel on lateral aspect of neural spine: absent (0); present (1). (X191, G191)

192. Anterior caudal vertebrae, transverse processes: ventral surface directed laterally or slightly ventrally (0); directed dorsally (1). (X192, G192)

193. Anterior caudal centra (excluding the first), articular face shape: amphiplatyan or amphicoelous (0); procoelous/distoplatyan (1); slightly procoelous (2); procoelous (3);posterior surface markedly more concave than the anterior one (4). (X193, G193)

194. Anterior caudal centra, pleurocoels: absent (0); present (1). (X194, G194)

195. Anterior caudal vertebrae, ventral surfaces: convex transversely (0); concave transversely (1). (X195, G195)

196. Anterior and middle caudal vertebrae, ventrolateral ridges: absent (0); present (1). (X196, G196)

197. Anterior and middle caudal vertebrae, triangular lateral process on the neural spine: absent (0); present (1). (X197, G197)

198. Anterior caudal transverse processes shape: triangular, tapering distally (0); "wing-like", not tapering distally (1). (X198, G198)

199. Anterior caudal neural spines, transverse breadth: approximately 50% of (0); orgreater than anteroposterior length (1). (X199, G199)

200. Anterior caudal transverse processes, proximal depth: shallow, on centrum only (0); deep, extending from centrum to neural arch (1). (X200, G200)

201. Anterior caudal transverse processes, diapophyseal laminae (ACDl, PCDL, PRDL, PODL): absent (0); present (1). (X201, G201)

202. Anterior caudal transverse processes, anterior centrodiapophyseal lamina (ACDL), shape: single (0); divided (1). (X202, G202)

203. Anterior caudal vertebrae, hyposphene ridge: absent (0); present (1). (X203, G203)

204. Anterior caudal centra, length: approximately the same (0); or doubling over the first 20 vertebrae (1). (X204, G204)

205. Anterior caudal neural arches, spinoprezygapophyseal lamina (SPRL): absent, or present as small short ridges that rapidly fade out into the anterolateral margin of the spine (0); present, extending onto lateral aspect of neural spine (1). X205, G205)

206. Anterior caudal neural arches, spinoprezygapophyseal lamina (SPRL)-spinopostzygapophyseal lamina (SPOL) contact: absent (0); present, forming a prominent lamina on lateral aspect of neural spine (1). (X206, G206)

207. Anterior caudal neural arches, prespinal lamina (PRSL): absent (0); present (1). (X207, G207)

208. Middle caudal centra, shape: cylindrical (0); with flat ventral margin (1); quadrangular, flat ventrally and laterally (2). (X208, G208)

209. Anterior and middle caudal centra, ventral longitudinal hollow: absent (0); present (1). (X209, G209)

210. Middle caudal centra, articular face shape: amphiplatyan or amphicoelous (0); procoelous/distoplatyan (1); slightly procoelous (2); procoelous (3). (X210, G210)

211. Middle caudal vertebrae, location of the neural arches: over the midpoint of the centrum with approximately subequal amounts of the centrum exposed at either end (0);on the anterior half of the centrum (1). (X211, G211)

212. Middle caudal vertebrae, height of the pedicels below the prezygapophysis: low with curved anterior edge of the pedicel (0); high with vertical anterior edge of the pedicel (1). (X212, G212)

213. Middle caudal vertebrae, orientation of the neural spines: anteriorly (0); vertical (1); slightly directed posteriorly (2); strongly directed posteriorly (3). (X213, G213)

214. Posterior caudal vertebrae, neural spine strongly displacced posteriorly: absent (0); present (1). (X214, G214).

215. Middle caudal vertebrae, ratio of centrum length to centrum height: less than 2, usually 1.5 or less (0); 2 or higher (1). (X215, G215)

216. Anterior-posterior caudal vertebrae (those with still well developed neural spine), neural spine orientation: vertical (0); slightly directed posteriorly (1); strongly directed posteriorly (2). (X216, G216)

217. Posterior Caudals centra, articular face shape: amphyplatic (0); procoelous (1); opisthocoelous (2). (X217, G217)

218. Posterior caudal centra, shape: cylindrical (0); dorsoventrally flattened, breadth at least twice height (1). (X218, G218)

219. Posterior caudal vertebrae, ratio of length to height: less than 5, usually 3 or less (0); 5or higher (1). (X219, G219)

220. Distalmost caudal centra, articular face shape: platycoelous (0); biconvex (1). (X220, G220)

221. Distalmost biconvex caudal centra, number: 10 or fewer (0); more than 30 (1) (X221, G221)

222. Distalmost biconvex caudal centra, length-to height ratio: less than 4 (0); greater than 5 (1). (X222, G222)

223. Forked chevrons with anterior and posterior projections: absent (0); present (1). (X223, G223)

224. Forked chevrons, distribution: distal tail only (0); throughout middle and posterior caudal vertebrae (1). (X224, G224)

225. Chevrons, crus bridging dorsal margin of haemal canal: present (0); absent (1). (X225, G225)

226. Chevron haemal canal, depth: short, approximately 25% (0); or long, approximately 50% chevron length (1). (X226, G226)

227. Chevrons: persisting throughout at least 80% of tail (0); disappearing by caudal 30 (1).(X227, G227)

228. Posterior chevrons, distal contact: fused (0); unfused (open) (1). (X228, G228)

229. Posture: bipedal (0); columnar, obligatory quadrupedal posture (1). (X229, G229)

230. Scapular acromion process, size: Narrow (0); broad, width more than 150% minimum width of blade (1). (X230, G230)

231. Scapular blade, orientation respect to coracoid articulation: perpendicular (0); forminga 45º angle (1). (X231, G231)

232. Scapular blade, shape: acromial edge not expanded (0); rounded expansion on acromial side (1); racquet-shaped (2). (X232, G232)

233. Scapula, acromion process dorsal margin: concave or straight (0); with V-shaped

concavity (1); with U-shaped concavity (2). (X233, G233)

234. Scapula, highest point of the dorsal margin of the blade: lower than the dorsal margin of the proximal end (0); at the same height than the dorsal margin of the proximal end (1); higher than the dorsal margin of the proximal end (2). (X234, G234)

235. Scapula, development of the acromion process: undeveloped (0); well developed (1). (X235, G235)

236. Scapular length/minimum blade breadth: 5.5 or less (0); 5.5 or more (1(X236, G236)

237. Scapula, ventral margin with a well developed ventromedial process: absent (0); present (1). (X237, G237)

238. Scapular, acromial process position: lies nearly glenoid level (0); lies nearly midpointscapular body (1). (X238, G238)

239. Scapular acromion length: less than 1/2 scapular length (0); at least 1/2 scapular length(1). (X239, G239)

240. Glenoid scapular orientation: relatively flat or laterally facing (0); strongly beveled medially (1). (X240, G240)

241. Scapular blade, cross-sectional shape at base: flat or rectangular (0); D-shaped (1). (X241, G241)

242. Coracoid, proximodistal length: less than the length of scapular articulation (0); approximately twice the length of scapular articulation (1). (X242, G242)

243. Coracoid, anteroventral margin shape: rounded (0); rectangular (1). (X243, G243)

244. Dorsal margin of the coracoid in lateral view: reaches or surpasses the the level of the dorsal margin of the scapular expansion (0); lies below the level of the scapular proximal expansion and separated from the latter by a V-shaped notch (1). (X244, G244)

245. Coracoid, Infraglenoid deep groove: absent (0); present (1). (X245, G245)

246. Coracoid, infraglenoid lip: absent (0); present (1). (X246, G246)

247. Sternal plate, shape: oval (0); crescentic (1). (X247, G247)

248. Prominent posterolateral expansion of the sternal plate producing a kidney-shaped profile in dorsal view: absent (0); present (1). (X248, G248)

249. Prominent parasagital oriented ridge on the dorsal surface of the sternal plate: absent (0); present (1). (X249, G249)

250. Ridge on the ventral surface of the sternal plate: absent (0); present (1). (X250, G250)

251. Ratio of maximum length of sternal plate to the humerus length: less than 0,75, usually less than 0,65 (0); greater than 0,75 (1). (X251, G251)

252. Humerus-to-femur ratio: less than 0.60 (0); 0.60 to 0.90 (1); greater than 0.90 (2). (X252, G252)

253. Humeral deltopectoral attachment, development: prominent (0); reduced to a low crest or ridge (1). (X253, G253)

254. Humeral deltopectoral crest, shape: relatively narrow throughout length (0); markedlyexpanded distally (1). (X254, G254)

255. Humeral midshaft cross-section, shape: circular (0); elliptical (1). (X255, G255)

256. Humerus, RI (sensu Wilson and Upchurch, 2003): Gracile (less than 0,27) (0); medium (0,28–0,32) (1); Robust (more than 0,33) (2). (X256, G256)

257. Humeral distal condyles, articular surface shape: restricted to distal portion of humerus (0); exposed on anterior portion of humeral shaft (1). (X257, G257)

258. Humeral distal condyle, shape: divided (0); flat (1). (X258, G258)

259. Humeral, lateral margin: medially deflected (0); almost straight until the half length oreven more (1). (X259, G259)

260. Humeral proximolateral corner, shape: rounded, the dorsal surface is well convex (0);

pronounced / square, the dorsal surface low, almost flat (1). (X260, G260)

261. Ulnar proximal condyle, shape: subtriangular (0); triradiate, with deep radial fossa (1). (X261, G261)

262. Ulnar proximal condylar processes, relative lengths: subequal (0); unequal, anterior arm longer (1). (X262, G262)

263. Ulnar olecranon process, development: prominent, projecting above proximal articulation (0); rudimentary, level with proximal articulation (1). (X263, G263)

264. Ulna, length-to-proximal breadth ratio: gracile (0); stout (1). (X264, G264)

265. Radial distal condyle, shape: round (0); subrectangular, flattened posteriorly and articulating in front of ulna (1). (X265, G265)

266. Radius, distal breadth: slightly larger than midshaft breadth (0); approximately twice midshaft breadth (1). (X266, G266)

267. Radius, distal condyle orientation: perpendicular to long axis of shaft (0); beveled approximately 20° proximolaterally relative to long axis of shaft (1). (X267, G267)

268. Carpal bones, number: 3 or more (0); 2 or fewer (1). (X268, G268)

269. Carpal bones, shape: round (0); block-shaped, with flattened proximal and distal surfaces (1). (X269, G269)

270. Metacarpus, shape: spreading (0); bound, with subparallel shafts and articular surfaces that extend half their length (1). (X270, G270)

271. Metacarpals, shape of proximal surface in articulation: gently curving, forming a 90arc (0); U-shaped, subtending a 270arc (1). (X271, G271)

272. Longest metacarpal-to-radius ratio: close to 0.3 (0); 0.45 or more (1). (X272, G272)

273. Metacarpal I, length: shorter than metacarpal IV (0); longer than metacarpal IV (1). (X273, G273)

274. Metacarpal I, distal condyle shape: divided (0); undivided (1). (X274, G274)

275. Metacarpal I distal condyle, transverse axis orientation: beveled approximately 20º respect to axis of shaft (0); proximodistally or perpendicular with respect to axis of shaft(1). (X275, G275)
276. Manual digits II and III, phalangeal number: 2-3-4-3-2 or more (0); reduced, 2-2-2-2-2or less (1); absent or unossified (2). (X276, G276)
277. Manual phalanx I.1, shape: rectangular (0); wedge-shaped (1). (X277, G277)
278. Manual nonungual phalanges, shape: longer proximodistally than broad transversely (0); broader transversely than long proximodistally (1). (X278, G278)
279. Pelvis, anterior breadth: narrow, ilia longer anteroposteriorly than distance separating preacetabular processes (0); broad, distance between preacetabular processes exceeds anteroposterior length of ilia (1). (X279, G279)
280. Ilium, ischial peduncle size: large, prominent (0); low, rounded (1). (X280, G280)
281. Ilium, dorsal margin shape: flat (0); semicircular (1). (X281, G281)
282. Ilium, preacetabular process shape: pointed, arching ventrally (0); semicircular, with posteroventral excursion of cartilage cap (1). (X282, G282)
283. Ilium, preacetabular process orientation: anterolateral to body axis (0); perpendicular to body axis (1). (X283, G283)
284. Highest point on the dorsal margin of the ilium: lies caudal to the base of the pubic process (0); lies cranial to the base of the pubic process (1). (X284, G284)
285. Pubis length respect to ischium: pubis slightly smaller or subequal to ischium (0); pubis larger (120% +) than ischium (1). (X285, G285)
286. Pubis, ambiens process development: small, confluent with anterior margin of pubis prominent, (0); projects anteriorly from anterior margin of pubis (1). (X286, G286)
287. Pubic apron, shape: flat (straight symphysis) (0); canted anteromedially (gentle S-shaped symphysis) (1). (X287, G287)

288. Puboischial contact, length: approximately one third total length of pubis (0); one-half total length of pubis (1). (X288, G288)

289. Ischium, acetabular articular surface: maintains approximately the same transverse width throughout its length (0); is transversely narrower in its central portion and strongly expanded as it approaches the iliac and pubic articulations (1). (X289, G289)

290. Ischium, iliac peduncle with constriction or "neck": absent (0); present (1). (X290, G290)

291. Ischium, elongate muscle scar on proximal end: absent (0); present (1). (X291, G291)

292. Ischial blade, shape: emarginate distal to pubic peduncle (0); no emargination distal to pubic peduncle (1). (X292, G292)

293. Ischia pubic articulation: less or equal to the anteroposterior length of pubic pedicel (0); greater than the anteroposterior length of pubic pedicel (1). (X293, G293)

294. Ischia, anteroposterior pubic pedicel width divided the total length of the ischium: lessthan 0,5 (0); 0,5 or grate (1); Large (2). (X294, G294)

295. Ischial distal shaft, shape: triangular, depth of ischial shaft increases medially (0); bladelike, medial and lateral depths subequal (1). (X295, G295)

296. Ischial distal shafts, cross-sectional shape: V-shaped, forming an angle of nearly 50º with each other (0); flat, nearly coplanar (1). (X296, G296)

297. Ischia, distal end: is only slightly expanded (0); is strongly expanded dorsoventrally (1). (X297, G297)

298. Ischium, angle formed between the shaft and the acetabular line: forming an almost right angle (80–110°) (0) or; a close angle (less than 70°) (1). (X298, G298)

299. Femur, fourth trochanter development: prominent (0); reduced to crest or ridge (1);

extremely reduced (2). (X299, G299)

300. Femur, lesser trochanter: present (0); absent (1). (X300, G300)

301. Femur midshaft, transverse diameter: subequal to anteroposterior diameter (0); 125-150% anteroposterior diameter (1); at least 185% anteroposterior diameter (2). (X301, G301)

302. Femur, lateral bulge (marked by the lateral expansion and a dorsomedial orientation of the laterodorsal margin of the femur, which starts below the femur head ventral margin):absent (0); present (1). (X302, G302)

303. Femur, pronounced ridge on posterior surface between greater trochanter and head: absent (0); present (1). (X303, G303)

304. Femur head position: perpendicular to the shaft, rises at the same level than the greatertrochanter (0); dorsally directed, rises well above the level of the greater trochanter (1). (X304, G304)

305. Femur, distal condyles relative transverse breadth: subequal (0); tibial much broader than fibular (1). (X305, G305)

306. Femur, distal condyles orientation: perpendicular or slightly beveled dorsolaterally (0); or beveled dorsomedially approximately 10 relative to femoral shaft (1). (X306, G306)

307. Femur, distal condyles articular surface shape: restricted to distal portion of femur (0);expanded onto anterior portion of femoral shaft (1). (X307, G307)

308. Situation of the femoral fourth trochanter: on the caudal surface of the shaft, near the midline (0); on the caudomedial margin of the shaft (1). (X308, G308)

309. Tibial proximal condyle, shape: narrow, long axis anteroposterior (0); expanded transversely, condyle subcircular (1). (X309, G309)

310. Tibial cnemial crest, orientation: projecting anteriorly (0); or laterally (1). (X310,

G310)

311. Tibia, distal breadth: approximately 125% (0); more than twice midshaft breadth (1). (X311, G311)
312. Tibial distal posteroventral process, size: broad transversely, covering posterior fossa of astragalus (0); shortened transversely, posterior fossa of astragalus visible posteriorly (1). (X312, G312)
313. Fibula, proximal tibial scar, development: not well-marked (0); well-marked and deepening anteriorly (1). (X313, G313)
314. Fibula, lateral trochanter: absent (0); present (1). (X314, G314)
315. Fibular distal condyle, size: subequal to shaft (0); expanded transversely, more than twice midshaft breadth (1). (X315, G315)
316. Astragalus, shape: rectangular (0); wedge shaped, with reduced anteromedial corner (1). (X316, G316)
317. Astragalus, fibular facet: faces laterally (0); faces posterolaterally, anterior margin visible in posterior view (1). (X317, G317)
318. Astragalus, foramina at base of ascending process: present (0); absent (1). (X318, G318)
319. Astragalus, ascending process length: limited to anterior two-thirds of astragalus (0); extending to posterior margin of astragalus (1). (X319, G319)
320. Astragalus, posterior fossa shape: undivided (0); divided by vertical crest (1). (X320, G320)
321. Astragalus, transverse length: 50% more than (0); or subequal to proximodistal height (1). (X321, G321)
322. Calcaneum: present (0); absent or unossified (1). (X322, G322)
323. Distal tarsals 3 and 4: present (0); absent or unossified (1). (X323, G323)

324. Metatarsus, posture: bound (0); spreading (1). (X324, G324)

325. Metatarsal I proximal condyle, transverse axis orientation: perpendicular to (0); angled ventromedially approximately 15º to axis of shaft (1). (X325, G325)

326. Metatarsal I distal condyle, transverse axis orientation: perpendicular to (0); angled dorsomedially to axis of shaft (1). (X326, G326)

327. Metatarsal I distal condyle, posterolateral projection: absent (0); present (1). (X327, G327)

328. Metatarsal I, minimum shaft width: less than that of metatarsals IIIV (0); or greater than that of metatarsals IIIV (1). (X328, G328)

329. Metatarsal I and V proximal condyle, size: smaller than (0); or subequal to those of metatarsals II and IV (1). (X329, G329)

330. Metatarsal III length: more than 30% (0); or less than 25% that of tibia (1). (X330, G330)

331. Metatarsals III and IV, minimum transverse shaft diameters: subequal to (0); or less than 65% that of metatarsals I or II (1). (X331, G331)

332. Metatarsal V, length: shorter than (0); or at least 70% length of metatarsal IV (1). (X332, G332)

333. Pedal nonungual phalanges, shape: longer proximodistally than broad transversely (0); broader transversely than long proximodistally (1). (X333, G333)

334. Pedal digits II–IV, penultimate phalanges, development: subequal in size to more proximal phalanges (0); rudimentary or absent (1). (X334, G334)

335. Pedal unguals, orientation: aligned with (0); or deflected lateral to digit axis (1). (X335, G335)

336. Pedal digit I ungual, length relative to pedaldigit II ungual: subequal (0); 25% larger than that of digit II (1). (X336, G336)

337. Pedal digit I ungual, length: shorter (0); or longer than metatarsal I (1). (X337, G337)

338. Pedal ungual I, shape: broader transversely than dorsoventrally (0); sickle-shaped, much deeper dorsoventrally than broad transversely (1). (X338, G338)

339. Pedal ungual IIIII, shape: broader transversely than dorsoventrally (0); sickle-shaped, much deeper dorsoventrally than broad transversely (1). (X339, G339)

340. Pedal digit IV ungual, development: subequal in size to unguals of pedal digits II and III (0); rudimentary or absent (1). (X340, G340)

341. Unguals of pedal digit II and III, proximal dimensions: as broad as deep (0); significantly broader than deep (1). (X341, G341)

342. Development of v-shaped wear facets in the teeth: well developed (forming ‘shoulders’) (0); slightly developed as marginal facets (1). (X342, G342)

343. Single planar wear facet in labial or lingual surface of the teeth: absent (0); present (1). (X343, G343)

344. One high angled wear facet and a second low angle wear facet in the teeth: absent (0);present (1). (X344, G344)

345. Tooth crown shape: narrow crowns (0); broad crowns (1). (X345, G345)

346. Middle to posterior dorsal vertebrae, pleurocoel dorsal margin: rounded (0) angular (1). (X346, G346)

347. Middle to posterior dorsal vertebrae, peurocoel dorsal margin: well below the dorsal margin of the centrum (0) at the level of the dorsal margin of the centrum or higher (1). (X347, G347)

348. Middle to posterior dorsal vertebrae, small fossa anterior to anteroventral to the pleurocoel: absent (0) present (1). (X348, G348)

349. Premaxilla-maxilla suture, shape: planar (0) twisted along its length, giving the

contact a sinuous appearance in lateral view (1). (X349, G349)

350. Premaxilla, small finger-like, vertically oriented premaxillary process near anteromedial corner of external naris: absent (0); present (1). (X350, G350)
351. Lacrimal, anteriorly projecting vertical plate of bone: absent (0); present (1). (X351, G351)
352. Dentary, posteroventral process shape: single (0) divided (1). (X352, G352)
353. Maxillary teeth, shape: straigth along axis (0); twisted axially through an arc of 30–45º(1). (X353, G353)
354. Axis, centrum shape: over two and a half times as long as tall (0); less than twice as long as tall (1). (X354, G354)
355. Cervical vertebrae, epipophyses shape: stout, pillar-like expansions above postzygapophyses (0); posteriorly projecting prongs (1). (X355, G355)
356. Middle and posterior cervical vertebrae, parapophyses shape: subcircular (0); elongate (1). (X356, G356)
357. Middle and posterior dorsal vertebral centra, keel: absent (0); present (1). (X357, G357)
358. Anterior caudal vertebrae (mainly the first and second): ventral bulge on transverse process: absent (0); present (1). (X358, G358)
359. Anterior and middle caudal vertebrae, blind fossae in lateral centrum: absent (0); present (1). (X359, G359)
360. Middle caudal vertebrae, transverse processes orientation: perpendicular (0); swept backwards, reaching the posterior margin of the centrum (1). (X360, G360)
361. Sternal plate, shape: posterolateral margin curved (0); posterolateral margin expanded as a corner (1). (X361, G361)
362. Humerus, strong posterolateral bulge on around level of the deltopectoral crest:

absent (0); present (1). (X362, G362)

363. Humerus, radial and ulnar condyles, shape: radial condyle divided on anterior face by a notch (0); undivided (1). (X363, G363)

364. Ilium, preacetabular process, kink on ventral margin: absent (0); present (1). (X364, G364)

365. Femur, longitudinal ridge on anterior face: absent (0); present (1). (X365, G365)

366. Fibula, proximal end, anterior crest: absent or poorly developed (0); well developed creating interlocking proximal cruz (1). (X366, G366)

367. Fibula, shaft shape: straight, or slightly sigmoidal (0); sigmoid, such that the proximal and distal faces are angled relative to midshaft (1). (X367, G367)

368. Astragalus, shape: at least 1.5 times wider than anteroposteriorly long (0); anteroposterior and transverse dimensions subequal (1). (X368, G368)

369. Metatarsal IV, proximomedial end, shape: flat or slightly concave (0); possesses a distinct embayment (1). (X369, G369)

370. Metatarsal IV, distal end, orientation: roughly perpendicular to long axis of bone (0); bevelled upwards medially (1). (X370, G370)

371. Exoccipital, dorsolateral margin in posterior view – spur of bone curves dorsolaterally and then ventrolaterally to form the dorsomedial margin of the posttemporal fenestra: absent (0); present (1). (X371, G371)

372. Exoccipital – small, deep, horizontally oriented groove immediately lateral to each of the proatlantal facets: absent (0); present (1) (new character). (X372, G372)

373. Postaxial cervical centra – small fossa on posteroventral corner of lateral surface: absent (0); shallow, anteroposteriorly elongate fossa present, posteroventral to main lateral pneumatic opening (1). (X373, G373)

374. Middle cervical neural spines – angle between PODL and SPOL in lateral view: acute

less than 85º (usually close to 45º) (0); 85º or more (usually 90º) (1). (X374, G374)

375. Posterior cervical and anterior dorsal bifid neural spines – morphology of metapophyses in anterior view: widely diverging (0) narrow, parallel to converging (1). (X375, G375)

376. Presacral neural spines, bifurcation: absent (0); present (1). (T126)

377. Cervical vertebrae, longitudinal sulcus on ventral surface: absent (0); present (1). (T133)

378. Mid- and posterior cervical vertebrae, pneumatization of lateral surface of centra: large, divided pleurocoel over approximately half of centrum (0); reduced, large fossa but sharp-bordered coel, if present, restricted to area above parapophysis (1). (T172)

379. Mid- and posterior cervical vertebrae, longitudinal ridge on ventral surface: present (0); absent (1). (T174)

380. Mid- and posterior cervical vertebrae, ventral keel: single (0); bifid, connects posterolaterally to the ventrolateral edges of the centrum (1); bifid, does not connect posterolaterally to the ventrolateral edges of the centrum (2). (Modified after T175) Modifications were introduced to adequately cover the variation in morphology, as in taxa such as *Tharosaurus* the ventral keel does not connect to the ventrolateral flanges.

381. Mid- and posterior cervical vertebrae, paired pneumatic fossae on ventral surface, separated by ventral midline keel: absent (0); present (1). (T176)

382. Mid- and posterior cervical centra with longitudinal flanges in the lateroventral edge on the posterior part of the centrum: absent (0); present (1). (T179)

383. Mid- and posterior cervical neural arches, interpostzygapophyseal lamina projects beyond the posterior margin of the neural arch (including the centropostzygapophyseal lamina), forming a prominent subrectangular projection in lateral view: absent (0); present (1). (T190)

384. Posterior cervical vertebrae, ventral keel: anteriorly placed (0); restricted to posterior portion of centrum (1); extending atleast upto the midlength of the centrum from the posterior margin (2). (Modified after T193)

Modifications were introduced to cover the variation in morphology, as in taxa such as *Tharosaurus* the ventral keel extends atleast upto the centrum midlength.

385. Posterior cervical neural arch, interpostzygapophyseal lamina (tpol): connects directly with roof of neural canal (0); vertical lamina connects tpol with neural canal roof (1). (T201)

386. Posterior bifid, cervical neural spines, medial surface: marked by distinct, dorsoventral ridge from base to spine summit (0); smooth (1). (T206)

387. Posterior cervical neural and/or anterior-most dorsal neural spines: vertical (0); anteriorly inclined (1) (T207)

388. Dorsal ribs, rib head: area between capitulum and tuberculum: flat (0); oblique ridge present that connects medial and lateral edge at the base of the rib head (1) (T283)

389. Anterior-most caudal centra, transverse cross-section: sub-circular with rounded ventral margin (0); ‘heart’-shaped with an acute ventral ridge (1) (T296)

390. Anterior caudal centra, pneumatopores: restricted to foramina (0); large coels present (1) (T307)

391. Mid-caudal vertebrae, lateral surface of centra: without longitudinal ridge at midheight (0); longitudinal ridge present, centra hexagonal in anterior/posterior view (1) (T333)

392. Mid- and posterior caudal vertebral centra, articular surfaces: subequal in width and height or higher than wide (0); considerably wider than high (1) (T342)

393. Mid- and posterior cervical vertebrae, pre-epipophysis: absent (0); present (1) (T181).

4. Supplementary Note 3: Phylogenetic analysis A1.

The phylogenetic affinity of *Tharosaurus* was tested against the expanded data matrix (75 taxa and 394 characters) of Gallina et al.[8] which included 21 diplodocoids, 35 macronarians and 19 non-neosauropods. As this dataset encompassed a spatiotemporally and phylogenetically diverse array of sauropodomorphs, it allowed for the placement of *Tharosaurus* anywhere within Sauropodamorpha. The phylogenetic analysis was performed in TNT version 1.6[10] where the software memory was set to retain 10000 trees with a display buffer of 10Mb (sensu Coria et al.[11]). Following Gallina et al.[8] the Traditional Search option was used to analyse the dataset. The constraints for the analysis included 5000 replications of Wagner trees, where the swapping algorithm was bisection reconnection and 10 trees were saved per replication. To determine the robustness of the nodes, Bremer support values were calculated using the script bremer.run where only tree suboptimal by 20 steps were retained. The outgroup taxon in this analysis was *Plateosaurus engelhardti*.

The analysis recovered 332 most parsimonious trees with a tree length of 1236, consistency index (CI) of 0.37 and retention index (RI) of 0.71. The strict consensus tree (Supplementary Fig. 5) corroborates previous studies[7,8]. The clade Sauropoda is well resolved with diplodocoids and macronarians showing distinct clustering within Neosauropoda. *Tharosaurus* is recovered as a dicraeosaurid flagellicaudatan, although the clade Dicraeosauridae is poorly resolved. In the 50% majority rule tree (Supplementary Fig. 6), Dicraeosauridae is better resolved where *Tharosaurus* is a sister taxon to ((*Pilmatueia* + *Amargatitanis*) + (*Brachytrachelopan* + (*Dicraeosaurus* + *Amargasaurus*))).

Tharosaurus shares four synapomorphies within Flagellicaudata — divided lateral pleuroceols on cervical centra (ch. 115); bifurcated presacral neural spines (ch. 376); lateroventral flanges on middle/posterior cervical centra (ch. 382); pre-epipophysis on middle/posterior cervicals (ch. 393)]. One unambiguous synapomorphy supports its recovery

as a dicraeosaurid — divided cprl in cervicals with the medial lamina connecting with the intraprezygapophyseal lamina (127). *Tharosaurus* also shares two synapomorphies with other diplodocoids — transversely concave ventral surface of cervical centra (ch. 112); quadrangular middle caudal centra (ch. 208). Furthermore, three autapomorphies characterize *Tharosaurus* — smooth and narrow prespinal lamina on non-bifid dorsal neural spine (ch 143), pleuroceol on anterior caudal (ch 194), ventral mid-line keel in middle/posterior cervicals bifurcating posteriorly by not meeting the lateroventral flanges (ch 380). Barring the last autapomorphy, the remaining are local, being also present in a few diplodocids.

4. Supplementary Note 4: Phylogenetic analysis A2.

The cervical neural spines are not preserved in *Tharosaurus*, but the neural arch morphology strongly suggests the presence of bifurcated spines. Consequently, analysis A2 was run testing the position of *Tharosaurus* with characters involving cervical spines scored as '?'.

The same methodology as stated above was followed which recovered 311 most parsimonious trees with a tree length of 1236, consistency index (CI) of 0.37 and retention index (RI) of 0.71. In both the strict consensus and majority-rule tree *Tharosaurus* is still recovered as a dicraeosaurid flagellicaudatan (Supplementary Figs. 7–8). In the majority-rule tree *Tharosaurus* is a sister taxon to ((*Pilmatueia* + *Amargatitanis*) + (*Brachytrachelopan* + (*Dicraeosaurus* + *Amargasaurus*))).

The recovery of *Tharosaurus* as a flagellicaudatan is supported by three synapomorphies — divided lateral pleuroceols on cervical centra (ch. 115), lateroventral flanges on middle/posterior cervical centra (ch. 382), and pre-epipophysis on middle/posterior cervicals (ch. 393). A single unambiguous synapomorphy support its recovery as a dicraeosaurid — divided cprl in cervicals with the medial lamina connecting with the intraprezygapophyseal lamina (127). Additionally, *Tharosaurus* also shares two synapomorphies with other diplodocoids — transversely concave ventral surface of cervical centra (ch. 112) and flat ventral margin surface of middle caudal centra articular surface (ch. 208).

The results of the alternative phylogenetic analysis corroborate the results of the taxonomic study as *Tharosaurus* is still recovered as a dicraeosaurid. Furthermore, *Tharosaurus* is phylogenetically bracketed by *Bajadasaurus* and ((*Pilmatueia* + *Amargatitanis*) + (*Brachytrachelopan* + (*Dicraeosaurus* + *Amargasaurus*))), all of which are

characterized by middle and posterior cervical vertebrae with bifurcated neural spines. Consequently, this allows for the inference of bifid neural spines in the middle/posterior cervical vertebrae of *Tharosaurs* and supports the finding of the osteological study.

3. Supplementary Table 1: List of specimens for the holotype of *Tharosaurus indicus* gen. et sp. nov.

Sl no.	Material	Registration number
1	Anterior cervical cotyle	RWR-241-A
2	Partial posterior cervical vertebra	RWR-241-B
3	Right prezygapophysis	RWR-241-C
4	Right prezygapophysis	RWR-241-D
5	Right prezygapophyseal articular surface	RWR-241-E
6	Partial anterior dorsal neural arch	RWR-241-F
7	Partial middle/posterior dorsal neural arch-spine complex	RWR-241-G
8	Partial middle/posterior dorsal neural arch-spine complex	RWR-241-H
9	Anterior dorsal rib	RWR-241-I
10	Partial anterior caudal vertebra	RWR-241-J
11	Middle caudal centrum	RWR-241-K

4. Supplementary Table 2: Measurement of skeletal specimens of *Tharosaurus indicus* gen. et sp. nov. All measurements are in mm; *c.* indicates estimated measurement. When added to the anatomical abbreviations, the suffix α indicates angle; H indicates height, L indicates length and W indicates width.

Reg no.	cH	cL	cW	nsα	nsL	nsW	przH	przL	przW	tpH	tpL	tpW
RWR-241-A	168.7	110.2										
RWR-241-B	165.8		210	59°	*c.*94.5	14.9						
RWR-241-C							124.3	51.6	41.6			
RWR-241-D							136.9	43.5	48.5			
RWR-241-E								41.5	57.7			
RWR-241-F										76.5	*c.78*.7	38.2
RWR-241-G					39.4	93.7				42.7	63.6	10.9
RWR-241-H					24	61.4						
RWR-241-J	136.4		113									
RWR-241-K	117.8	161.3	74.4									

Abbreviations: c, centrum; ns, neural spine; prz, prezygapophysis; tp, transverse process. Measured parameters shown in Supplementary Fig. 2.

Supplementary Figures

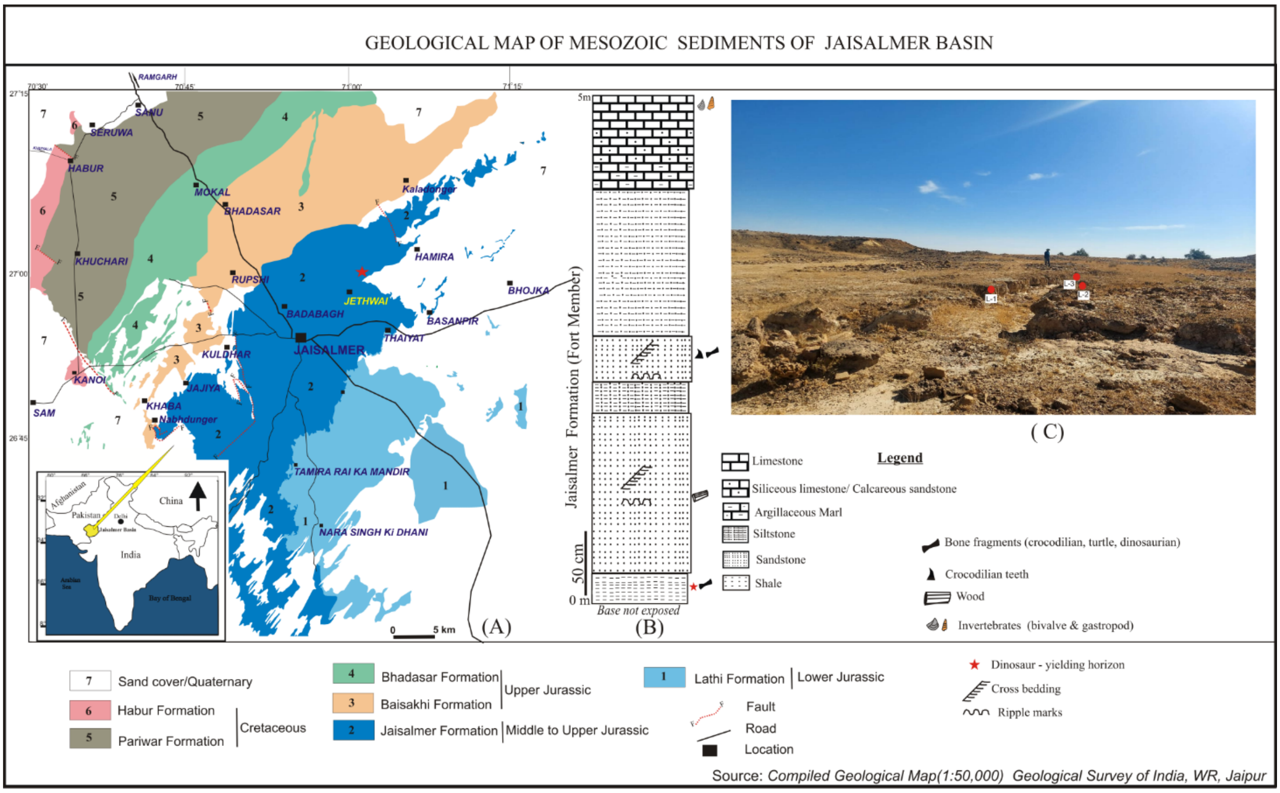

Figure. 1 Geological map of Jaisalmer Basin showing (**a**) the fossil locality; (**b**) stratigraphic column showing the position of the dinosaur fossil yielding horizon; (**c**) photograph of the fossil site. The map and stratigraphic column are drawn by K.K. using CorelDRAW 2019 (Version number: 21.0.0.593, URL link: http://www.corel.com/en/)

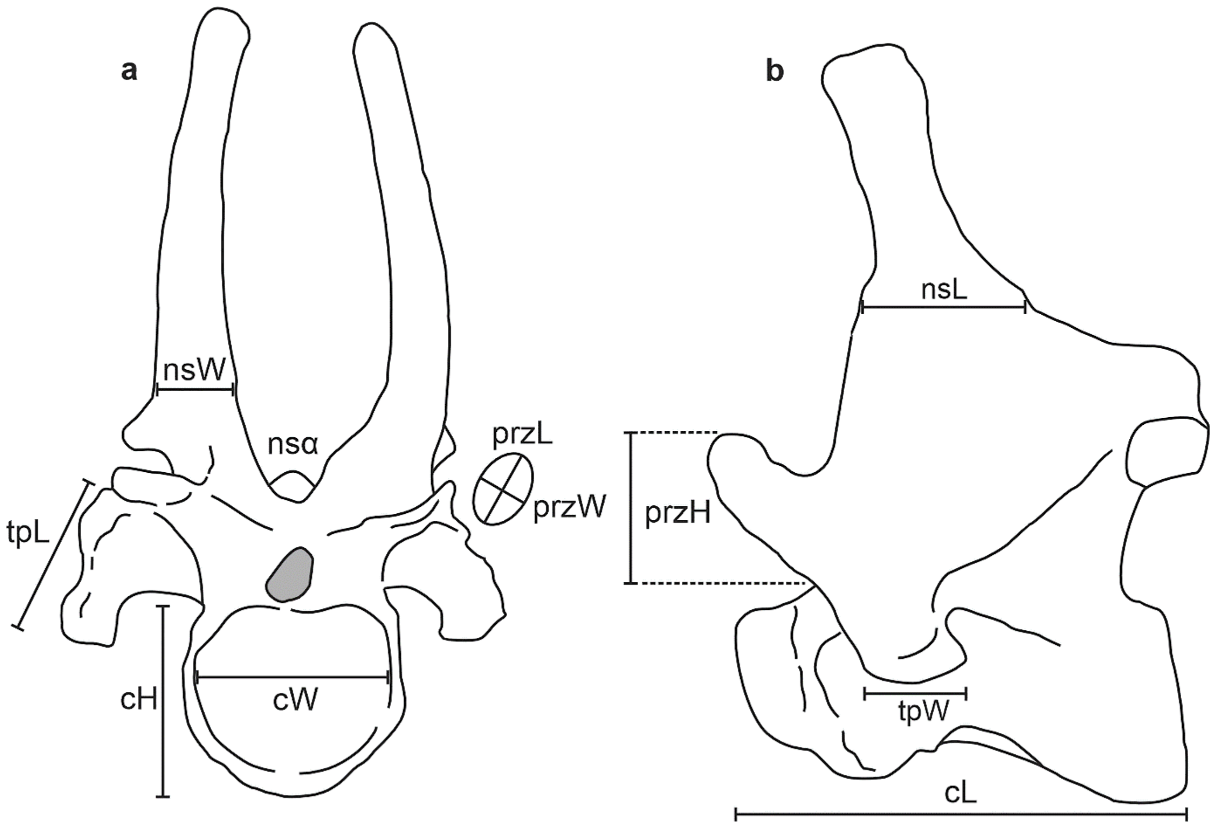

Supplementary Fig. 2. Index of measured parameters shown on the schematic representation of dicraeosaurid vertebra in: anterior (a) and lateral (b) views. When added to the anatomical abbreviations, the suffix α indicates angle; H indicates height, L indicates length and W indicates width. Abbreviations: c, centrum; ns, neural spine; prz, prezygapophysis; tp, transverse process. Schematic diagrams not to scale and after Coria *et al*.[11]

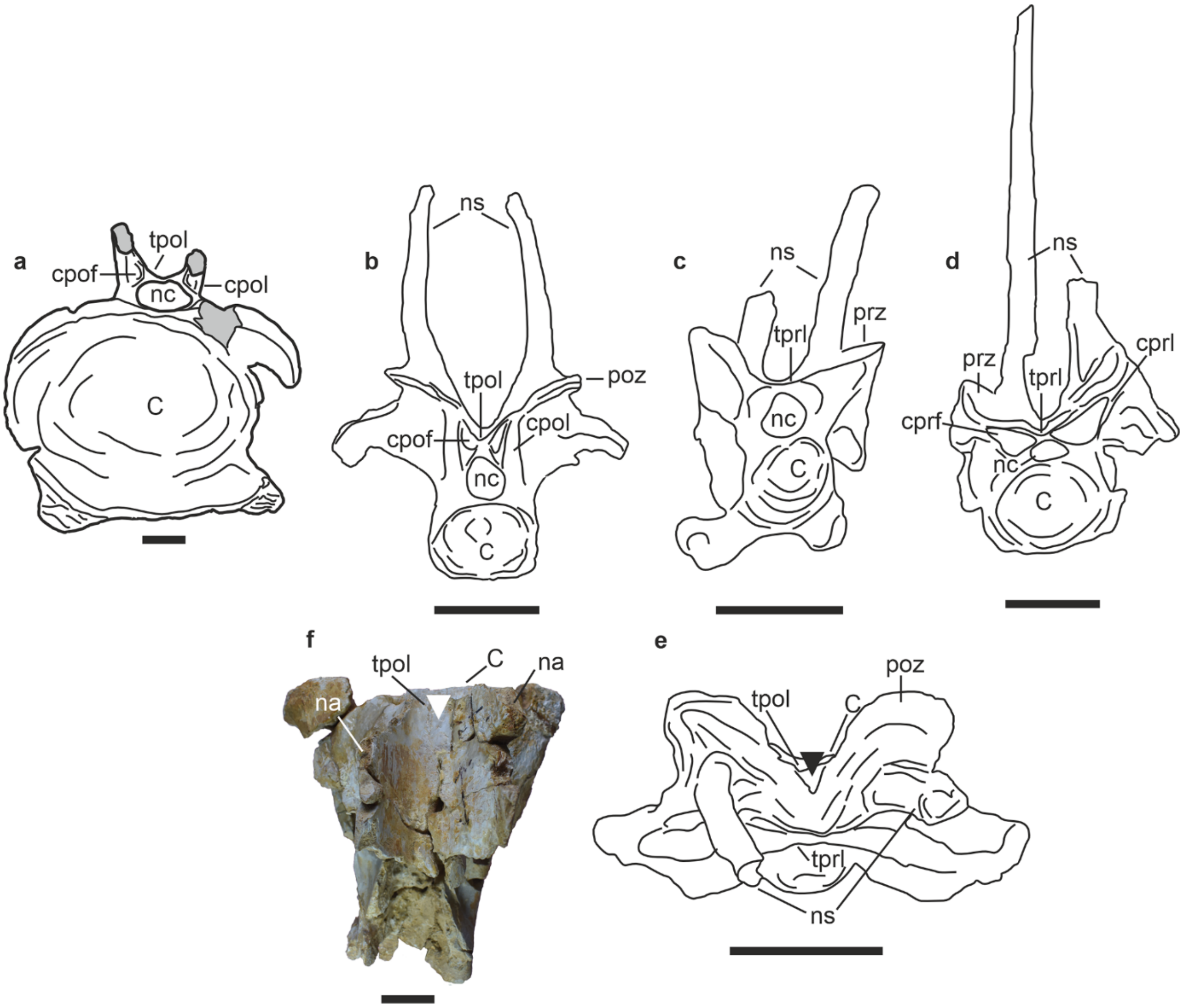

Supplementary Fig. 3. Comparison of *Tharosaurus indicus* gen. et sp. nov. with other dicraeosaurids. Middle/posterior cervical vertebra (RWR-241-B) of *Tharosaurus* in posterior view (**a**); cervico-dorsal vertebra (MLL-Pv-002) of *Pilmatueia faundezi* in posterior view (line drawing after Coria *et al.*[11]) (**b**); middle cervical vertebra of *Amargasaurus* (MACN PV N15) in anterior view (line drawing after Windholz *et al.*[12]) (**c**); cervicodorsal vertebra of *Amargasaurus* (MACN PV N15) in anterior view (line drawing after Windholz *et al.*[12]) (**d**); middle/posterior cervical vertebra (RWR-241-B) of *Tharosaurus* in dorsal view (actual photograph) (**e**); cervico-dorsal vertebra (MLL-Pv-002) of *Pilmatueia faundezi* in dorsal view (line drawing after Coria *et al.*[11]) (**f**). Arrow heads indicate passage enclosed by deeply bifurcated neural arches. Abbreviations: c, centrum; cpof, centropostzygapophyseal fossa; cpol, centropostzygapophyseal lamina; cprf, centroprezygapophyseal fossa; cprl,

centroprezygapophyseal lamina; na, neural arch; nc, neural canal; ns, neural spine; poz, postzygapophysis; prz, prezygapophysis; tpol, intrapostzygapophyseal lamina; tprl, intraprezygapophyseal lamina. Scales bars represent 50 mm (a, e) and 100 mm (b–d, f).

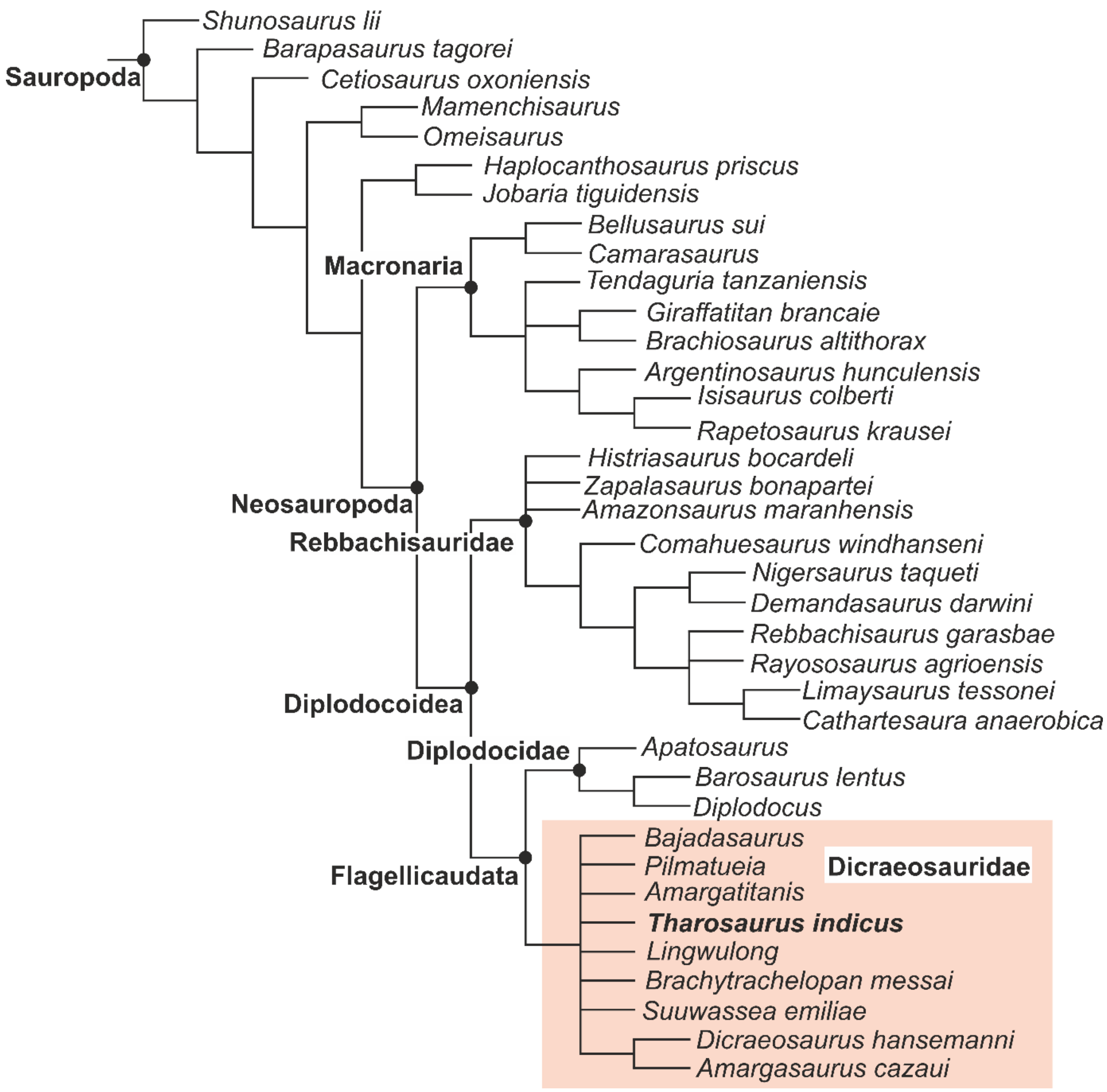

Supplementary Fig. 4. Phylogenetic position of *Tharosaurus indicus* gen. et sp. nov. (RWR-241) in strict consensus tree. Clade Dicraeosauridae shaded in pink.

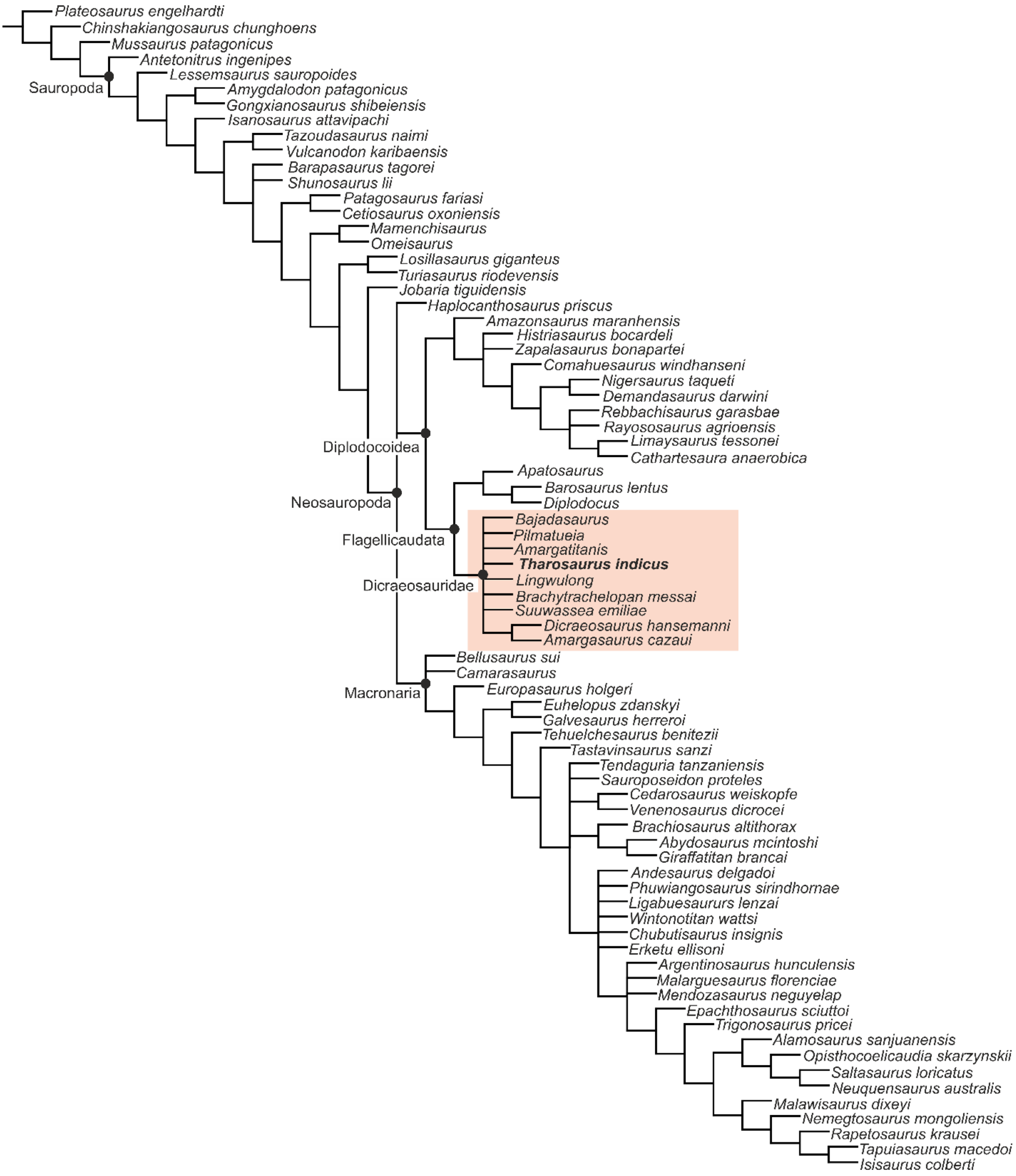

Supplementary Fig. 5. Strict consensus tree of phylogenetic analysis A1 showing position of *Tharosaurus indicus* gen. et sp. nov. (RWR-241). Clade Dicraeosauridae shaded in pink.

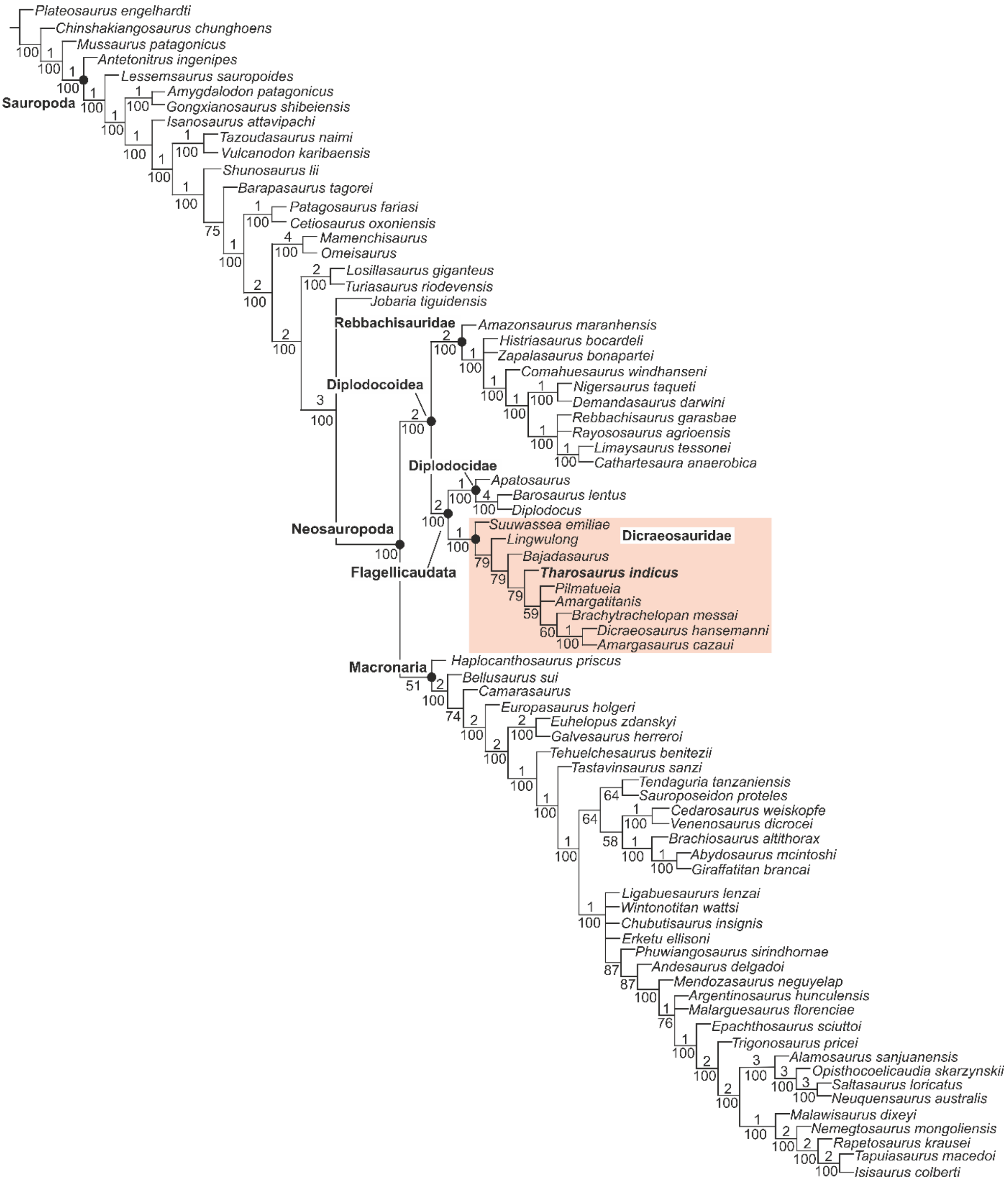

Supplementary Fig. 6. 50% majority-rule tree of phylogenetic analysis A1 showing position of *Tharosaurus indicus* gen. et sp. nov. (RWR-241). Clade Dicraeosauridae shaded in pink. Numbers above nodes indicate Bremer support values.

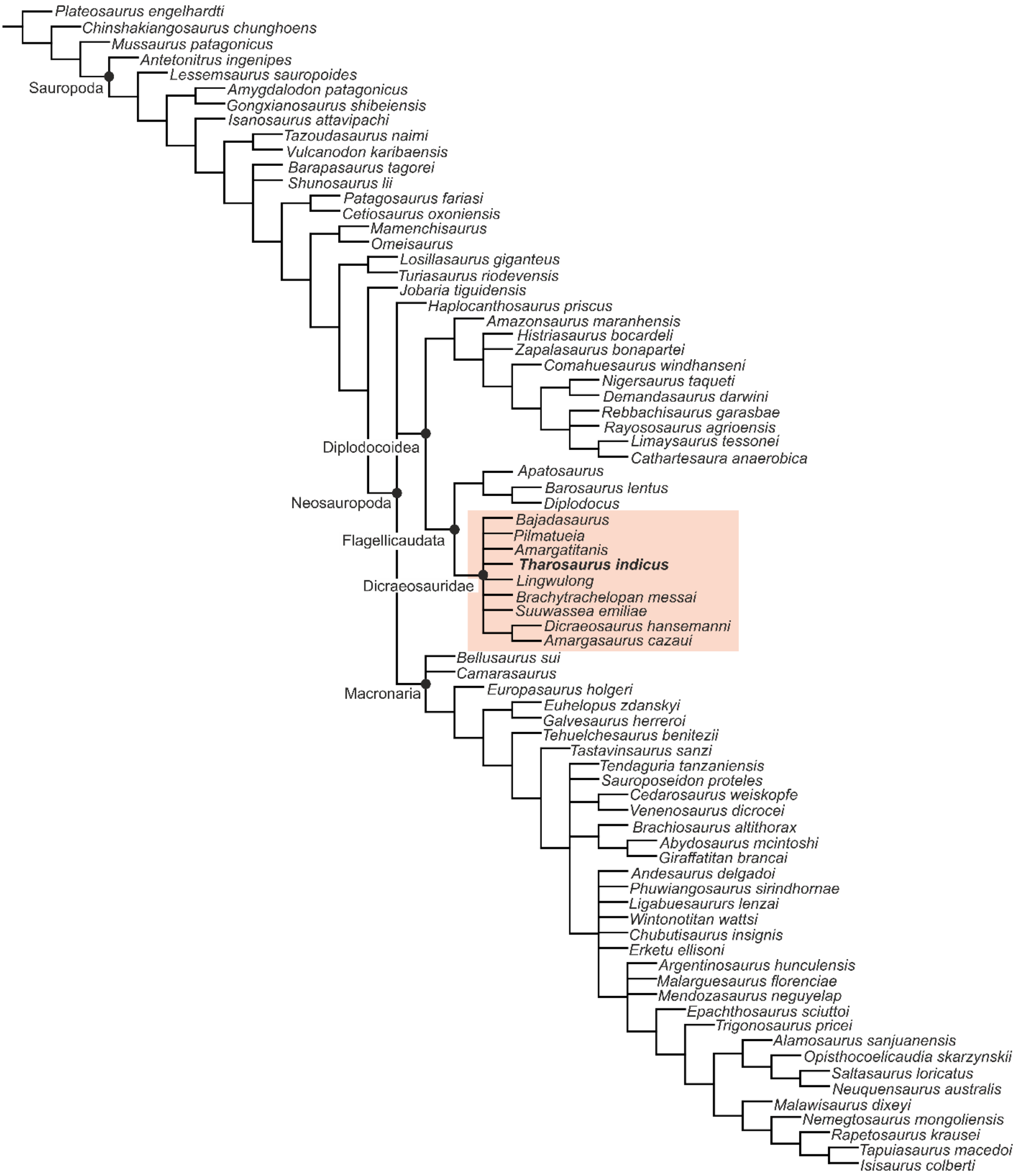

Supplementary Fig. 7. Strict consensus tree of phylogenetic analysis A2 showing position of *Tharosaurus indicus* gen. et sp. nov. (RWR-241). Clade Dicraeosauridae shaded in pink.

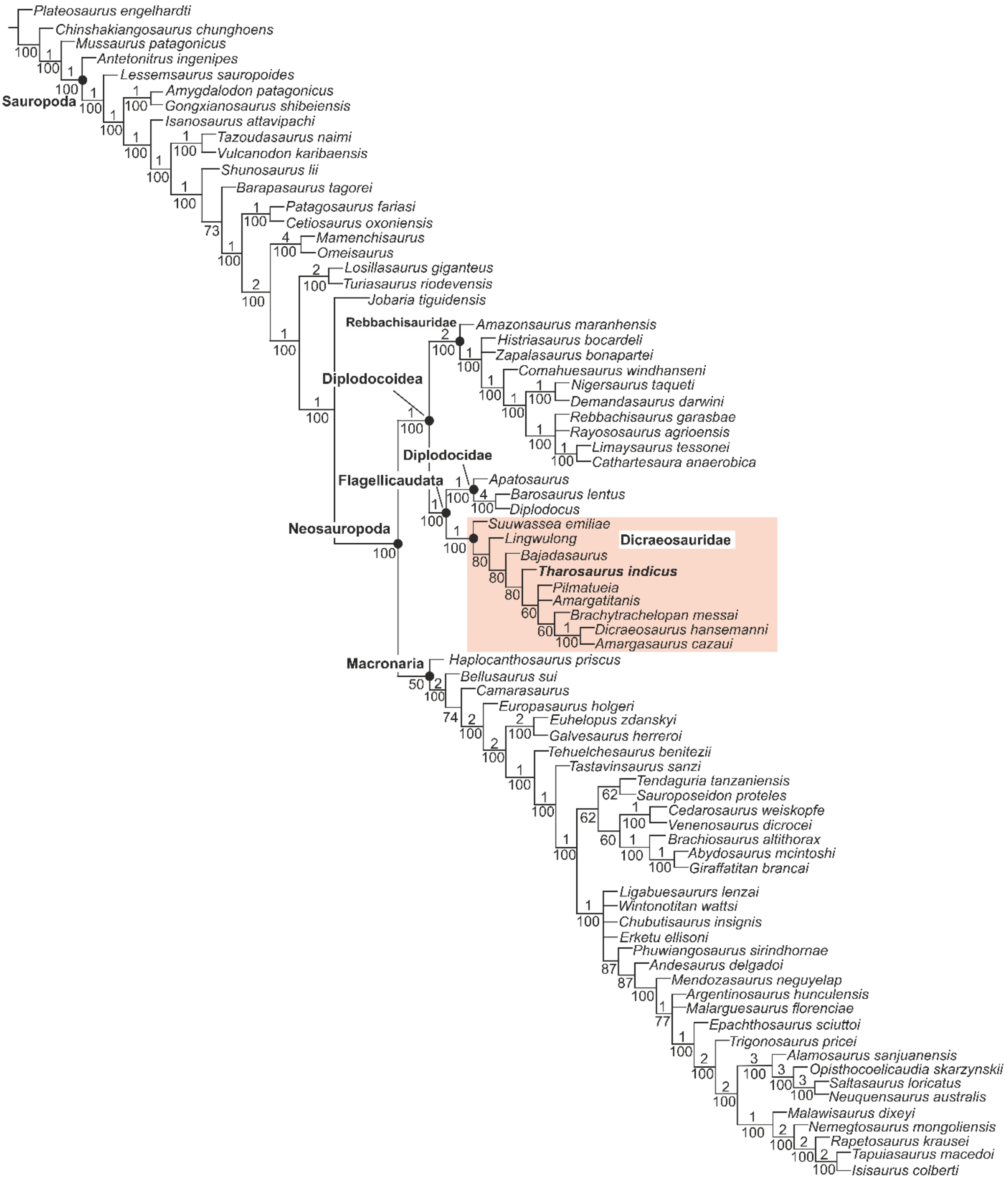

Supplementary Fig. 8. 50% majority-rule tree of phylogenetic analysis A2 showing position of *Tharosaurus indicus* gen. et sp. nov. (RWR-241). Clade Dicraeosauridae shaded in pink. Numbers above nodes indicate Bremer support values.

Supplementary References

1. Pandey, D.K., Choudhary. S., Bahadur, T., Swami, N. & Sha, J. A review of the Lower - lowermost Upper Jurassic lithostratigraphy of the Jaisalmer Basin, western Rajasthan, India - an implication on biostratigraphy. *Vol. Jurass.* **10**, 61–82 (2012).
2. Sharma, A., Hendrickx, C. & Singh, S. First Theropod record from the marine Bathonian of Jaisalmer Basin, Tethyan coast of Gondwanan India. *Riv. Ital. di Paleontol. e Stratigr.* **129**, 49–64 (2023).
3. Kumar, K., Bajpai, S., Pandey, P., Ghosh, T. & Bhattacharya, D. Hybodont sharks from the Jurassic of Jaisalmer, western India. *Hist. Biol.* **34**, 953–963 (2022).
4. Dasgupta SK. 1975. A revision of the Mesozoic-Tertiary stratigraphy of the Jaisalmer basin, Rajasthan. *Ind. J. Earth Sci.* **2**, 77–94.
5. Kumar, K., Bajpai, S., Ghosh, T. Pandey, P. & Bhattacharya, D. Oldest East Gondwanan pycnodont fishes (Neopterygii, Pycnodontiformes) from the Middle Jurassic (Bathonian) of Jaisalmer, western India. *PalZ*, **96**, 795–804 (2022).
6. Pandey, D.K., Fürsich, F.T. & Sha, J. Interbasinal marker intervals—A case study from the Jurassic basins of Kachchh and Jaisalmer, western India. *Sci. China Series D: Earth Sci.* **52**, 1924–1931 (2009).
7. Xu, X. *et al.* A new Middle Jurassic diplodocoid suggests an earlier dispersal and diversification of sauropod dinosaurs. *Nat. Comm.* **9**, 2700 (2018).
8. Gallina, P. A., Apesteguía, S. & Canale, J. I. A new long-spined dinosaur from Patagonia sheds light on sauropod defense system. *Sci. Rep.* **9**, 1392 (2019).
9. Tschopp, E., Mateus, O. & Benson, R. B. A specimen-level phylogenetic analysis and taxonomic revision of Diplodocidae (Dinosauria, Sauropoda). *PeerJ*, **3**, e857 (2015).
10. Goloboff, P.A. & Morales, M. E. TNT version 1.6, with a graphical interface for MacOS and Linux, including new routines in parallel. *Cladistics*, **39**, 144–153 (2023).

11. Coria, R. A., Windholz, G. J., Ortega, F. & Currie, P. J. A new dicraeosaurid sauropod from the lower cretaceous (Mulichinco formation, Valanginian, Neuquén Basin) of Argentina. *Cretac*. *Res*. **93**, 33–48 (2019).

12. Windholz, G. J., Carballido, J. L., Coria, R. A., Zurriaguz, V. L. & Rauhut, O. W. 2023. How pneumatic were the presacral vertebrae of dicraeosaurid (Sauropoda: Diplodocoidea) dinosaurs? *Biol*. *J*. *Linn*. *Soc*. **138**, 103–120.
